# Effect of End Groups on the Cloud Point Temperature of Aqueous Solutions of Thermoresponsive Polymers: An Inside View by Flory–Huggins Theory

**DOI:** 10.3390/polym16040563

**Published:** 2024-02-19

**Authors:** Thi To Nga Dang, Erik Nies

**Affiliations:** Department of Chemistry, KU Leuven, Celestijnenlaan 200F, 3001 Leuven, Belgium; erik.nies@kuleuven.be

**Keywords:** thermoresponsive polymers, end groups, cloud point temperature, lower critical solution behavior, dilute solution regime, FH theory for end-functionalized homopolymers

## Abstract

In an effort to gain insight into the origin of the effects of end groups on the cloud point temperature ($T_{cp}$) as a function of the polymer molar mass of thermoresponsive polymers with lower critical solution behavior in dilute aqueous solutions, we use the Flory–Huggins (FH) theory amended for end groups. The theory was applied to available experimental data sets of poly(N-isopropylacrylamide) (PNIPAM), poly(4-vinylbenzyl methoxytris(oxyethylene) ether) (PTEGSt), and poly(*α*-hydro-*ω*-(4-vinylbenzyl)tetrakis(oxyethylene) ether) (PHTrEGSt). The theory relates the variations in $T_{cp}\left(M,\phi_{cp}\right)$ for different end groups to the *effective* FH χ parameter of the end groups and explains the qualitative notion that the influence of the end groups is related to the hydrophobicity/hydrophilicity of the end groups relative to that of the so called intrinsic $T_{cp}\left(M,\phi_{cp}\right)$ response of a polymer without end groups. The limits to the applicability of the FH theory are established, and a set of possible theoretical improvements is considered. The ultimate scrutiny of the simple FH theory and suggested improved theories must await the measurement of truly thermodynamic cloud points; the available cloud points are merely estimations of the thermodynamic cloud point, for which the deviation to the true cloud point cannot be established with sufficient accuracy.

## 1. Introduction

Thermoresponsive polymers have been well known for many decades as a class of “smart” and advanced materials thanks to their ability to respond to changes in temperature [1,2,3]. Such polymers are soluble in water at low temperatures and exhibit liquid–liquid (L-L) phase separation with increasing temperature, known as a lower critical solution temperature (LCST) phase separation. This generic property has been used in a wide range of applications, including sensing technology [4,5], surface modification [6,7], and biomedical applications [8,9,10,11].

In practical applications of thermoresponsive polymers with LCST, it is convenient and versatile that the cloud point temperature ($T_{cp}$), a commonly studied parameter to characterize the miscibility behavior, can be tuned not only by varying the polymer molar mass (distribution), the chemical nature of repeat units, the architecture, etc. [12,13,14,15,16], but also through the modification of the end groups of so-called *α*, *ω*-end-functionalized polymers [17,18,19,20,21,22,23,24]. Turbidity measurements often serve as a practical tool to determine an estimate of the true $T_{cp}$ [25]. Although numerous studies have been conducted to investigate the effect of end groups on $T_{cp}$, the acquired results are not yet fully understood.

$T_{cp}$ *Dependence on End Group at Fixed Molar Mass in (Sufficiently) Dilute Solution:*

It was reported that $T_{cp}$, for a given polymer molar mass, can either increase [23,26], decrease [17,20,21], or hardly be affected [19] by the end group(s) introduced. The hydrophilicity/hydrophobicity of end groups has been employed in the qualitative interpretation of the obtained results. Particularly, end groups that are more hydrophilic compared to polymer repeat units tend to increase $T_{cp}$ by enhancing polymer–water interactions. In contrast, hydrophobic end groups tend to decrease $T_{cp}$ by enhancing polymer–polymer interactions. Moreover, neutral end groups, i.e., end groups that are almost identical or very similar to polymer repeat units, show no or minor influences on $T_{cp}$_._ The sometimes unexpected invariance of $T_{cp}$ in *α*, *ω*-end-functionalized polymers has also been explained by the effective cancellation of the opposite effects on $T_{cp}$ of the hydrophilic and hydrophobic end groups in the *α* and *ω* positions of the polymer chain [27]. Additionally, the absolute magnitude of a given end group effect is most pronounced at small polymer molar masses, reducing with increasing molar masses [17,20,24,28].

$T_{cp}$ *Dependence on Molar Mass for Given End Group in (Sufficiently) Dilute Solution:*

It was also reported that the incorporation of an end group can result either in an inverse dependence [13,17,24], a direct dependence [18,20,24,28,29,30], or an independence [24,27,31] of $T_{cp}$ on the polymer molar mass. In agreement with the studies discussed in the previous paragraph, the effect of end groups is more noticeable at small polymer molar masses and tends to diminish with increasing molar mass. Moreover, the inverse dependence of $T_{cp}$ on the polymer molar mass is considered to be in agreement with the Flory–Huggins (FH) theory for (homo)polymer solutions, in which this effect is directly a consequence of the reduced combinatorial entropy from mixing with increasing molar mass, leading to a reduced miscibility with increasing molar mass of the polymer in solutions [32,33,34,35,36,37].

In sufficiently dilute solutions, the direct dependence of $T_{cp}$ on the polymer molar mass was attributed to the presence of very hydrophobic end groups that strongly reduce the miscibility of the polymer in water due to the unfavorable interactions between the end groups and water, and thus the $T_{cp}$ decreases [30]. This effect is stronger for lower polymer molar masses since, for solutions of identical polymer concentrations, the concentration of the end groups is larger in solutions of lower polymer molar masses.

$T_{cp}$ *Dependence on End Group and Molar Mass at Higher Polymer Concentrations:*

At higher polymer concentrations, it was reported that very hydrophobic end groups can associate due to their poor solubility in water, resulting in the formation of polymer aggregates in solutions below $T_{cp}$, causing an increase in the *apparent* polymer molar mass (measured, e.g., by static light scattering) and a decrease in the entropy of mixing, resulting in the L-L phase separation of the solution at lower temperatures, and the effect is the strongest for the shortest polymers [24,30,38].

The effect of hydrophobic end groups can also be eliminated by micelle formation, with the hydrophobic end groups as the core being isolated from the solvent and the polymer chains as the shell. For instance, in PNIPAM, the occurrence of micelle formation has been observed and studied in great detail [26,27,39,40].

$T_{cp}$ *and FH Theory:*

The conventional FH theory for homopolymer solutions has been successfully used to explain the inverse dependence of $T_{cp},$ with an increase in the polymer molar mass in polymer solutions showing LCST L-L phase separation [32,33,34,35,36,37]. However, the observed independence of $T_{cp}$ or the direct dependence of $T_{cp}$ on the molar mass observed for certain end groups cannot be explained by the conventional FH homopolymer solution theory.

With the aim of gaining a further understanding of the effect of end groups, an alternative interpretation of the variation in $T_{cp}$ with the end groups was investigated, employing the FH theory amended for end groups. In the following sections, we first introduce the thermodynamic definition of the cloud point and present the governing FH equations for an idealized homopolymer and for a more realistic homopolymer with end groups in the framework of the FH theory. Subsequently, the method utilized to extract end-group-related parameters from molar-mass-related parameters for further investigation is presented. We then apply our model to available experimental data sets, PNIPAM, PTEGSt, and PHTrEGSt, to demonstrate the applicability of the theoretical model.

In the Discussion, we elaborate on the theoretical predictions of the simple FH theory for end-functionalized homopolymers and its capability to rationalize the experimentally observed variations in cloud point data with variations in molar masses and end groups. In addition, the applicability limits of the simple FH theory and a set of possible theoretical improvements to the simple theory are considered. To conclude, we sum up our findings, suggest improvements that could be considered for the experimental determination of cloud point data, and reflect briefly on their application to systems beyond aqueous solutions of thermoresponsive polymers.

## 2. FH Theory Amended for End Groups

### 2.1. General Thermodynamics of Mixtures

The general conditions for the heterogeneous L-L phase equilibrium in a multi-component mixture containing n components are as follows:

(1) $$\begin{array}{l} T^{\prime }=T^{\prime \prime }\;(\mathrm{thermal}\mathrm{equilibrium})\\ p^{\prime }=p^{\prime \prime }\;(\mathrm{mechanical}\mathrm{equilibrium})\\ \Delta \mu_{i}^{\prime }=\Delta \mu_{i}^{\prime \prime }\;(\forall i\;\mathrm{present}\mathrm{in}\mathrm{both}\mathrm{coexisting}\mathrm{phases})\;(\mathrm{chemical}\mathrm{equilibrium}) \end{array}$$

where the single and double prime superscripts denote the respective coexisting liquid phases and $\Delta \mu_{i}$ is the excess chemical potential of component i, which can be calculated from the excess Gibbs energy of mixing, $\Delta_{mix}G$, according to the standard thermodynamic definition: $\Delta \mu_{i}={\left.\frac{\partial {∆}_{mix}G}{\partial N_{i}}\right|}_{T,p,\forall N_{j}\neq N_{i}},$ with $N_{i\left(j\right)}$ being the number of molecules of component $i\left(j\right)$.

For a one-phase homogenous liquid solution of a binary mixture of a monodisperse polymer in a single solvent, $T_{cp}$ of this solution at a fixed mixture composition and constant pressure is the temperature at which the very first sign of L-L phase separation is observed. The general conditions (Equation (1)) can be cast into a set of two equations, as follows:

(2) $$\begin{array}{l} \Delta \mu_{O}\left(\phi_{P}^{\prime },T,p\right)=\Delta \mu_{O}\left(\phi_{P}^{\prime \prime },T,p\right)\;\mathrm{and}\\ \Delta \mu_{P}\left(\phi_{P}^{\prime },T,p\right)=\Delta \mu_{P}\left(\phi_{P}^{\prime \prime },T,p\right) \end{array}$$

with $\Delta \mu_{O}$ $\left(\Delta \mu_{P}\right)$ being the excess chemical potential of the solvent (polymer) and $\phi_{P}^{\prime }$ and $\phi_{P}^{\prime \prime }$ being the polymer concentrations in the coexisting phases.

The thermodynamic $T_{cp}$ is obtained by solving Equation (2) for $T_{cp}$ and the composition$,\;\phi_{P}^{\prime \prime },$ of the incipient phase that is in equilibrium with the composition $\phi_{P}^{\prime }=\phi_{P}$, the latter being the fixed composition of the homogeneous starting solution.

The formulae in Equation (2) are exact, and if we had an exact theory for $\Delta_{mix}G$, the exact answer would also be obtained. Unfortunately, the theories are approximate, and the solution will also be approximate. Nevertheless, a theoretical analysis can give insight into the causes of the observed dependences of the cloud point on, e.g., the molar mass and end groups.

### 2.2. The FH Theory and the Cloud Point of a Homopolymer

To start the theoretical analysis, we consider the seminal FH expression for the excess Gibbs energy of the mixing of a monodisperse homopolymer [32,33,34,35]. (The end groups and middle units of the polymer are the same, which in reality is evidently strictly impossible).

(3) $$\frac{\Delta_{mix}G_{FH\;}}{N_{L}k_{B}T}=\phi_{O}\ln\phi_{O}+\frac{\phi_{P}}{s_{P}}\ln\phi_{P}+\chi \phi_{O}\phi_{P}$$

with the first two terms on the right-hand side representing the combinatorial excess entropy of the mixing polymer and the solvent and the last term expressing the excess enthalpy of mixing, $\phi_{O}$ *(*$\phi_{P})$ being the volume fraction of the solvent (polymer), $N_{L}$ the total number of lattice sites, $k_{B}$ the Boltzmann constant, $s_{P}$ the number of lattice sites taken by the polymer molecule, and χ the FH interaction parameter of the solvent–polymer combination. In Equation (3), it is explicitly assumed that the solvent molecule occupies one lattice site; thus, the solvent molecule volume sets the lattice site volume, $v_{l}$, defining also the volume taken up by a polymer segment on the lattice, which is, in general, not equal to the volume of the chemical polymer repeat unit. (From now on, the term “segment” will be used instead of “segment on the lattice” to denote in the theory the repeat entity on the lattice to measure the size of the real polymer molecule). The terms repeat unit, middle unit, and end group will refer to the chemical moieties of the real molecule.

The FH interaction parameter is, according to the original FH theory, only dependent on temperature, according to $\chi =\frac{\chi_{1}}{T}$, where $\chi_{1}=\frac{z}{k_{B}}\left(\epsilon_{OP}-\frac{\left(\epsilon_{PP}+\epsilon_{OO}\right)}{2}\;\right)$ is a system-specific parameter with the dimensions of temperature [K], z is the lattice coordination number, and $\epsilon_{OP},\;\epsilon_{PP},and\;\epsilon_{OO}$ are the characteristic interaction energies of the solvent–polymer segment, polymer segment–polymer segment, and solvent–solvent pairs, respectively.

The excess chemical potentials are given by

(4) $$\frac{\Delta \mu_{O}}{k_{B}T}=\ln\phi_{O}+\phi_{P}\left(1-\frac{1}{s_{P}}\right)+\chi \phi_{P}^{2}\;\mathrm{and}\;\frac{\Delta \mu_{P}}{k_{B}Ts_{P}}=\frac{1}{s_{P}}\ln\phi_{P}+\phi_{O}\;\left(\frac{1}{s_{P}}-1\right)+\chi \phi_{O}^{2}$$

It is well known that the theoretical temperature dependence of χ is not sufficient to describe experimental data, and it is widespread practice to use extended temperature-dependent, $\chi \left(T\right),$ functions. Equation (5) combines the most commonly used temperature-dependent, $\chi \left(T\right),$ interaction functions:

(5) $$\chi =\chi_{0}+\frac{\chi_{1}}{T}+\chi_{2}T+\chi_{3}\ln\left(T\right)$$

With $\chi_{i}$ being constants. Thermodynamic and molecular arguments for the functional form of $\chi \left(T\right)$ are available [41,42,43]. In the work to follow, we will use $\chi =\chi_{0}+\frac{\chi_{1}}{T}$.

### 2.3. The FH Theory Amended for a Polymer with End Groups

It was soon observed that the end groups of a polymer chain influenced the thermophysical properties of the polymer. This influence should also be reflected in the excess Gibbs energy of mixing. An *α*, *ω*-end-functionalized homopolymer can be viewed as a particular case of a copolymer. An FH type of theory for copolymers was developed alongside the FH theory of a homopolymer solution (Section 2.2). Employing the same approximations that give rise to $\Delta_{mix}G_{FH}$ for the homopolymer for a statistical (*A*,*B*)-copolymer with two different repeat units, *A* and *B*, leads to the conclusion that the excess Gibbs energy of mixing is also given by Equation (3) with the stipulation that the FH interaction parameter is now dependent on the *copolymer composition* and the combinatorial entropy of mixing is not [44,45,46,47,48].

Here, an *α*, *ω*-end-functionalized homopolymer is theoretically treated in the FH spirit as a polymer containing middle and end segments. Each distinguishable copolymer molecule contributes the typical combinatorial entropy of a mixing term to the Gibbs energy of the mixing expression, which is identical to that of a homopolymer with the same number of segments. The effect of the end groups is only present in the enthalpic contribution to the excess Gibbs energy of mixing in an *effective* FH interaction function, depending *only* on the copolymer composition. For an *α*, *ω*-end-functionalized monodisperse homopolymer of molar mass M, the *effective* FH interaction function is directly related to the molar mass of the *α*, *ω*-end-functionalized monodisperse polymer. Accordingly, the FH expression for the excess Gibbs energy of mixing for an *α*, *ω*-end-functionalized monodisperse homopolymer in solution is also given by Equation (3), but with χ given by (see Appendix A.1)

(6) $$\begin{array}{l} \chi =\chi_{OM}\left(1-\frac{M_{E}}{M}\right)+\chi_{OE_{1}}\left(\frac{n_{E_{1}}M_{E_{1}}}{M}\right)-\chi_{ME_{1}}\left(1-\frac{M_{E}}{M}\right)\left(\frac{n_{E_{1}}M_{E_{1}}}{M}\right)\\ \;\;\;\;\;\;\;+\chi_{OE_{2}}\left(\frac{n_{E_{2}}M_{E_{2}}}{M}\right)-\chi_{ME_{2}}\left(1-\frac{M_{E}}{M}\right)\left(\frac{n_{E_{2}}M_{E_{2}}}{M}\right)\\ \;\;\;\;\;\;\;-\chi_{E_{1}E_{2}}\left(\frac{n_{E_{1}}M_{E_{1}}}{M}\right)\left(\frac{n_{E_{2}}M_{E_{2}}}{M}\right) \end{array}$$

where $\chi_{IJ}$ is the FH pair interaction parameter between type *I* and type *J* segments ($\chi_{OM},\;\chi_{OE_{1}},\;\chi_{OE_{2}},\;\chi_{ME_{1}},\;\chi_{ME_{2}},\;\mathrm{and}\;\chi_{E_{1}E_{2}}$ are the FH pair interaction parameters of, respectively, the solvent–middle segment pair, the solvent–end segment pairs, the middle segment–end segment pairs, and the end segment–end segment pair) and $M_{E}=n_{E_{1}}M_{E_{1}}+n_{E_{2}}M_{E_{2}},$ where $M_{E_{i}}$ is the molar mass of the end group of type i, and the molar mass of the linker that connects the end group with the polymer repeat unit is also included in $M_{E_{i}}$. Note that $n_{E_{i}}$ is either 0 (no end group of type $E_{i}$ present) or 1 (end group of type $E_{i}$ is present). In the FH approximation, $\chi_{IJ}=\frac{\chi_{IJ,1}}{T}$, but, just as in the case of the homopolymer mentioned in Section 2.2, the interaction parameters can have extra temperature dependencies, as given by Equation (5).

Equations (3) and (6) have the same dependence on the mixture composition, and the effects of the intramolecular details are only contained within the χ parameter. Therefore, the chemical potentials are given by Equation (4), with χ given in Equation (6).

The original homopolymer FH theory is recovered from the *α*, *ω*-end-functionalized homopolymer FH theory in several limiting cases:

- Case 1A: $n_{E_{1}}=n_{E_{2}}=0$, i.e., distinct end segments are not present, and the homopolymer FH theory in its original form is recovered exactly;
- Case 1B: $s_{P}\to \;\infty$; in this limit, the influence of distinct end segments vanishes, and the original homopolymer FH theory is recovered exactly;
- Case 2A: a homopolymer with two end groups, $E_{1}$ and $E_{2},$ with $n_{E_{1}}$ and $n_{E_{2}}=1$ exactly recovers the original homopolymer FH theory when $\chi_{OM}=\chi_{OE_{1}}=\chi_{OE_{2}}$ and $\chi_{ME_{1}}=\chi_{ME_{2}}=\chi_{E_{1}E_{2}}=0$.
- Case 2B: A homopolymer with two end groups, $E_{1}$ and $E_{2},$ with $n_{E_{1}}$ and $n_{E_{2}}=1$ gives very similar results as the original homopolymer FH theory; in fact, this is the most realistic case as all real polymers have end groups differing from the middle units. However, when the interaction functions of the solvent with the end groups and the middle units are very similar, their influence is also identical, and the original homopolymer FH theory is, in practice, recovered. This happens when $\chi_{OM}≅\chi_{OE_{1}},\;\chi_{OM}≅\;\chi_{OE_{2}}$ and $\chi_{ME_{1}},\chi_{ME_{2}},\chi_{E_{1}E_{2}}≅0$ or when the contributions of the end groups effectively cancel, i.e., $\left(-\chi_{OM}\left(\frac{M_{E}}{M}\right)+\chi_{OE_{1}}\left(\frac{M_{E_{1}}}{M}\right)-\chi_{ME_{1}}\left(1-\frac{M_{E}}{M}\right)\left(\frac{M_{E_{1}}}{M}\right)+\chi_{OE_{2}}\left(\frac{M_{E_{2}}}{M}\right)-\chi_{ME_{2}}\left(1-\frac{M_{E}}{M}\right)\left(\frac{M_{E_{2}}}{M}\right)-\chi_{E_{1}E_{2}}\left(\frac{M_{E_{1}}}{M}\right)\left(\frac{M_{E_{2}}}{M}\right)\right)≅0$.

### 2.4. Thermodynamic Definition of the Cloud Point, $\chi_{cp}$

By using Equations (2) and (4) at a fixed composition, $\phi_{cp},$ varying $s_{P},$ and solving for $\phi_{P}^{\prime \prime }$ and $\chi_{cp}$ instead of *T*, the generic dependence of $\chi_{cp}$ and $\phi_{P}^{\prime \prime }$ on $s_{P}$ is obtained.

**Figure 1:**
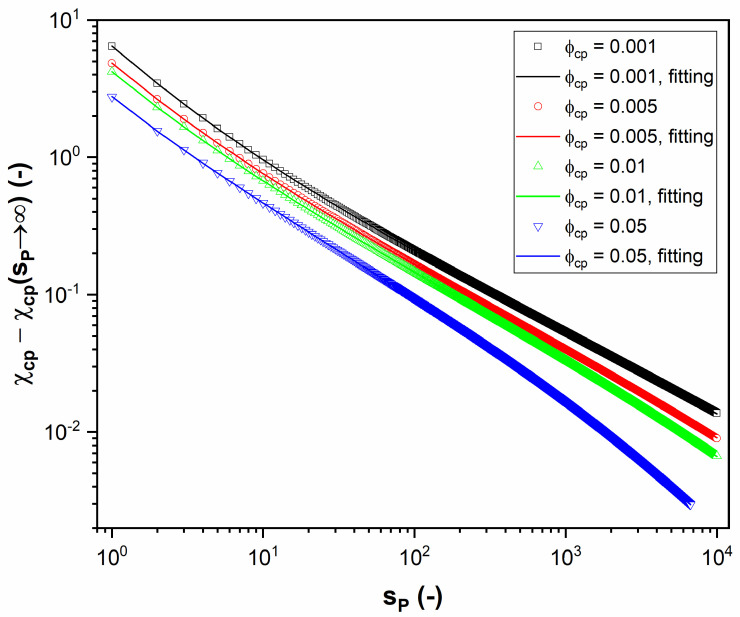
Double logarithmic plot of $\chi_{cp}\left(\phi_{cp},\;s_{P}\right)-\chi_{cp}\left(\phi_{cp},\;s_{P}\to \infty \right)$ versus $s_{P}$ according to the FH theory (Equations (2) and (4)) for selected compositions, $\phi_{cp}$ (symbols), and the empirical fit of the data using Equation (7) (solid lines).

In Figure 1, $\chi_{cp}\left(\phi_{cp},\;s_{P}\right)-\chi_{cp}\left(\phi_{cp},\;s_{P}\to \;\infty \right)$ is presented versus $s_{P}$ for $\phi_{cp}=0.05,\;0.01,\;0.005,\;\mathrm{and}\;0.001$. In the limit of $s_{P}\to \infty$ and for any finite $\phi_{cp}$, the coexisting phase composition $\phi_{P}^{\prime \prime }=0$ and the equilibrium $\chi_{cp}\left(s_{P}\to \;\infty \right)=-\frac{\left(\phi_{cp}+\ln\left(1-\phi_{cp}\right)\right)}{\phi_{cp}^{2}}$ are presented in Table 1.

The exact solutions of the coexistence condition in Equations (2) and (4) were, with sufficient accuracy, reproduced in Equation (7):

(7) $$\left(\chi_{cp}\left(\phi_{cp},s_{P}\right)-\chi_{cp}\left(\phi_{cp},s_{P}\to \infty \right)\right)=10^{\left(\overset{6}{\underset{i=0}{\sum }}B_{i}{\left(\log s_{P}\right)}^{i}\right)}$$

Equation (7) is convenient to use in the further analysis of the experimental data, and for selected $\phi_{cp},$ the coefficients $B_{i}$ in Equation (7) are summarized in Table 1.

### 2.5. Separating the Influences of End Segments and Middle Segments

For further analysis (see Section 3), it is convenient to separate in Equation (6) the effect of the end segments $\left(\Delta \chi_{E}\right)$ from the middle segments as follows:

(8) $$\begin{array}{l} \Delta \chi_{E}\equiv \chi -\chi_{OM}\left(1-\frac{M_{E}}{M}\right)=\chi_{OE_{1}}\left(\frac{M_{E_{1}}}{M}\right)-\chi_{ME_{1}}\left(1-\frac{M_{E}}{M}\right)\left(\frac{M_{E_{1}}}{M}\right)+\chi_{OE_{2}}\left(\frac{M_{E_{2}}}{M}\right)-\chi_{ME_{2}}\left(1-\frac{M_{E}}{M}\right)\left(\frac{M_{E_{2}}}{M}\right)-\\ \;\chi_{E_{1}E_{2}}\left(\frac{M_{E_{1}}}{M}\right)\left(\frac{M_{E_{2}}}{M}\right)=a_{1}\left(\frac{1}{M}\right)+a_{2}{\left(\frac{1}{M}\right)}^{2} \end{array}$$

with $a_{1}=\left(\chi_{OE_{1}}-\chi_{ME_{1}}\right)M_{E_{1}}+\left(\chi_{OE_{2}}-\chi_{ME_{2}}\right)M_{E_{2}}$ and $a_{2}=\left(\chi_{ME_{1}}M_{E}M_{E_{1}}+\chi_{ME_{2}}M_{E}M_{E_{2}}-\chi_{E_{1}E_{2}}M_{E_{1}}M_{E_{2}}\right)$.

For polymers with one end group of type $E_{1}$, Equation (8) reduces to

(9) $$\Delta \chi_{E_{1}}=\chi_{OE_{1}}\left(\frac{M_{E_{1}}}{M}\right)-\chi_{ME_{1}}\left(1-\frac{M_{E_{1}}}{M}\right)\left(\frac{M_{E_{1}}}{M}\right)=\left(\chi_{OE_{1}}-\chi_{ME_{1}}\right)\left(\frac{M_{E_{1}}}{M}\right)+\chi_{ME_{1}}{\left(\frac{M_{E_{1}}}{M}\right)}^{2}=a_{1}\left(\frac{1}{M}\right)+a_{2}{\left(\frac{1}{M}\right)}^{2}$$

with $a_{1}=\left(\chi_{OE_{1}}-\chi_{ME_{1}}\right)M_{E_{1}}$ and $a_{2}=\chi_{ME_{1}}{\left(M_{E_{1}}\right)}^{2}$.

The advantage of Equations (8) and (9) is that the right-hand side only depends on the properties of the end group(s) and is, at most, a quadratic function of the reciprocal molar mass of the polymer.

### 2.6. Theoretical Predictions of $T_{cp}$ for a Hypothetical α-End-Functionalized Homopolymer

Using the *α*, *ω*-end-functionalized homopolymer theory presented by Equations (4), (6), and (7) combined with $\chi \left(T\right)=\chi_{0}+\chi_{1}/T$ (the minimal requirement to describe the LCST), we can already illustrate that the theory is able to reproduce the typical $T_{cp}$ versus M dependencies found in the experimental systems. As an example, the *α*-end-functionalized homopolymer is represented on the lattice by $s_{P}$ segments, of which one is an end segment of type $E_{1}$. The solvent, middle, and end segments each take one lattice site. For the solvent–middle segment pair, we set $\chi_{OM}\left(T\right)=5-\frac{1350}{T},$ and for the solvent–end segment pair, we vary $\chi_{OE_{1}}\left(T\right)=5+\frac{\chi_{OE_{1},1}}{T},$ making the interaction of the solvent–end segment energetically more (less) favorable compared to the interaction of the solvent–middle segment when $\chi_{OE_{1},1}<-1350\;K\;\left(\chi_{OE_{1},1}>-1350\;K\right)$. All the remaining parameters in Equation (6) are set equal to zero (i.e., $\chi_{ME_{1}}=0$ and $n_{E_{2}},\chi_{OE_{2}},\;\chi_{ME_{2}},\;\chi_{E_{1}E_{2}}=0)$.

With this choice, Equation (6) simplifies to $\chi \left(T\right)=\chi_{OM}\left(1-\frac{1}{s_{P}}\right)+\chi_{OE_{1}}\left(\frac{1}{s_{P}}\right)=\chi_{OM}\left(T\right)+\frac{\left(\chi_{OE_{1},1}-\chi_{OM,1}\right)}{s_{P}T}$.

**Figure 2:**
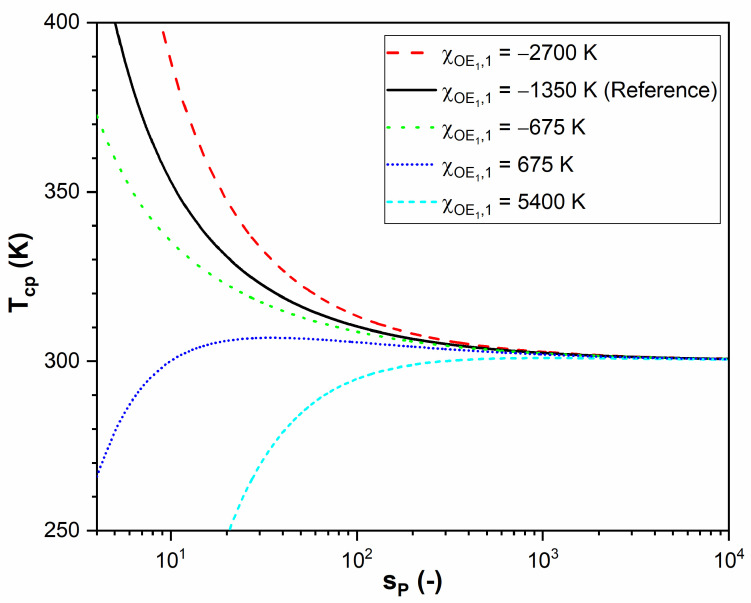
The influence of $\chi_{OE_{1},1}$ of the end segment on $T_{cp}$ of a polymer solution $\left(\phi_{P}=0.01\right)$ as a function of the polymer chain length, $s_{P}$.

The predicted $T_{cp}$s for $\phi_{cp}=0.01$ are shown as a function of $s_{P}$ in Figure 2.

- $\chi_{OE_{1},1}=-1350\;K:$ the curve is the result for a polymer that has no distinct end segments (Case 1A).
- $\chi_{OE_{1},1}=-2700\;K:$ the end segment, compared to the middle segment, has a more favorable interaction with the solvent, and a steeper cloud point curve is obtained.
- $\chi_{OE_{1},1}=-675\;K:$ the end segment, compared to the middle segment, has a less favorable interaction with the solvent, and the cloud point curve falls off more gently with $s_{P}$.
- $\chi_{OE_{1},1}=+675\;K:$ compared to the middle segment, the interaction of the solvent with the end segment worsens further; $T_{cp}$ initially increases with $s_{P}$, reaches a maximum, and subsequently reaches the asymptotic value $s_{P}\to \;\infty$, where the effect of the end group can be ignored.
- $\chi_{OE_{1},1}=+5400\;K$: $T_{cp}$ increases asymptotically without a maximum to its limiting value at $s_{P}\to \;\infty$.

Finally, all the cloud point curves converge asymptotically in the limit of $s_{P}\to \;\infty$ to the same value, $T_{cp}^{\infty }\left(\phi_{cp}\right)$.

## 3. Application of FH Theory to Available Experimental Data

### 3.1. Selected Systems

The *α*, *ω*-end-functionalized homopolymer FH theory will be used to analyze and interpret the influence of end groups on the experimental $T_{cp}$ data of systems available in the literature. The experimental systems were selected based on proper information on the molecular characterization of polymers synthesized using controlled polymerization routes that yield relatively narrow molar mass distributions and control over the introduced end groups. Such polymers have indeed been made with the purpose of systematically studying the effect of molar mass and the end groups on the cloud point of the thermoresponsive polymers. These systems were already described in the Introduction and, as mentioned there, interpreted using concepts like the hydrophilic/hydrophobic nature of the end groups relative to the middle unit. The systems that are presented are PNIPAM, PTEGSt, and PHTrEGSt.

All experimental data are available in the original references either as tabulated data or in the figures; in the latter case, the data points were digitized from figures using Origin 2018b [49]. Detailed information concerning the molar masses, dispersity, and $T_{cp}$s is available in the Supplementary Materials, Tables S1–S3.

### 3.2. Relationships between Experimental Data and Theoretical Parameters

In the FH theory, the parameters are $\phi_{P},\;s_{P},\;\chi_{IJ,}$ and $M_{E_{i}}$. The necessary relationships with the experimental characteristics are as follows:

(10) $$s_{P}=M/\rho_{P}V_{l}=M_{n}/\rho_{P}V_{l}$$

where $\rho_{P}$ is the mass density of the polymer and $V_{l}$ is the molar volume of the lattice site, which is equal to the molar volume of the solvent, i.e., $V_{l}\equiv V_{O}=M_{O}/\rho_{O}$. For water, we take $V_{l}=18$ cm^3^/mol and $\rho_{0}=1$ g/cm^3^, assuming $\rho_{P}=1$ g/cm^3^. This assumption seems reasonable for all the polymers considered here. This choice of densities is not essential for the predictions of the theory, and when accurate density data are available, they can be used instead. The FH theory Equations (3)–(9) are only valid for a monodisperse polymer in a solvent. The studied polymer samples have a relatively narrow molar mass distribution, and we will treat them as monodisperse; hence, in the second equality in Equation (10), we set $M=M_{n}$.

The simplest FH interaction function that can describe LCST miscibility behavior is $\chi_{IJ}=\chi_{IJ,0}+\chi_{IJ,1}/T$. This is not the only choice possible. For all experimental data, the temperature range, ΔT, covered is, in most cases, very small, ΔT≅20 K at most, and $\chi_{IJ}=\chi_{IJ,0}+\chi_{IJ,1}\times T$ would describe the experimental data equally well. However, we prefer the first choice as $\chi_{IJ,0}$ $\left(\chi_{IJ,1}\right)$ are of pure entropic (energetic) origin, respectively. Moreover, the introduction of distinct end groups automatically adds additional parameters, $\chi_{IJ}$, that must assume values. The addition of $n_{e}$ distinct end groups requires $\left(n_{e}+2\right)\left(n_{e}+1\right)/2-1$ additional $\chi_{IJ}$-functions, and for $\chi_{IJ}=\chi_{IJ,0}+\chi_{IJ,1}/T$, this would require $2\left(\left(n_{e}+2\right)\left(n_{e}+1\right)/2-1\right)$ parameter values to represent the experimental data. Therefore, for an *α*, *ω*-end-functionalized homopolymer, $n_{e}=2$ and 10 additional parameters, $\chi_{IJ,k},$ must be set. In view of the limited data (typically at most three to seven cloud point temperatures), it is even impossible to determine all these parameters’ values, as we have more parameters than experimental data. The limited set of data points per end-functionalized polymer as well as the anticipated limited accuracy of the experimental $T_{cp}$s hamper the level of detail in χ that can really be extracted. Nevertheless, it will become clear that we still can extract useful information on the effect of end groups on $T_{cp}$.

### 3.3. PNIPAM

**Scheme 1:**
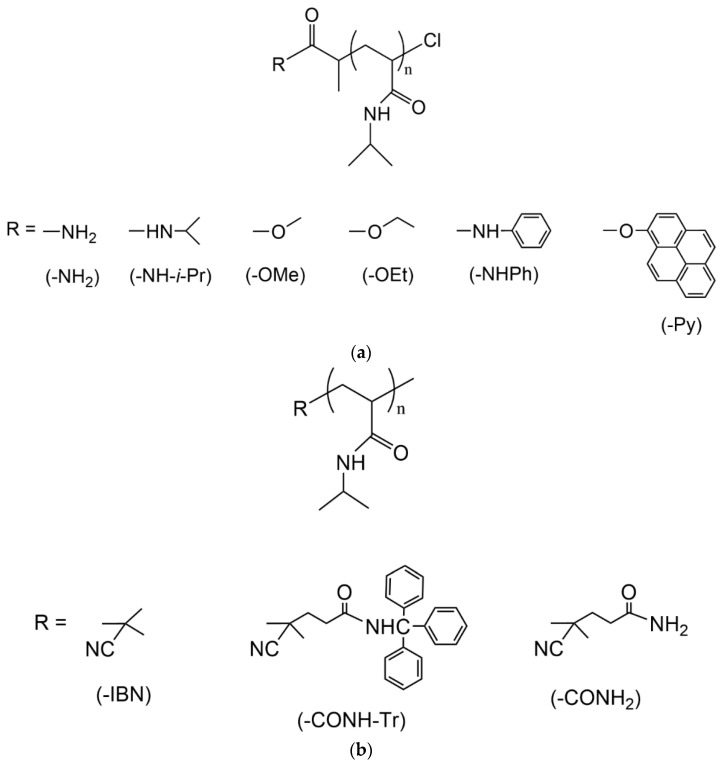
(**a**) Narrow-disperse RCOCHCH_3_-(NIPAM)_n_-Cl with R= -NH_2_, -NH-*i*-Pr, -OEt, -OMe, -NHPh [13,17] and R= -Py [18]; (**b**) R-(NIPAM)_n_- with R= -IBN, -CONH-Tr, -CONH_2_ [29].

**Figure 3:**
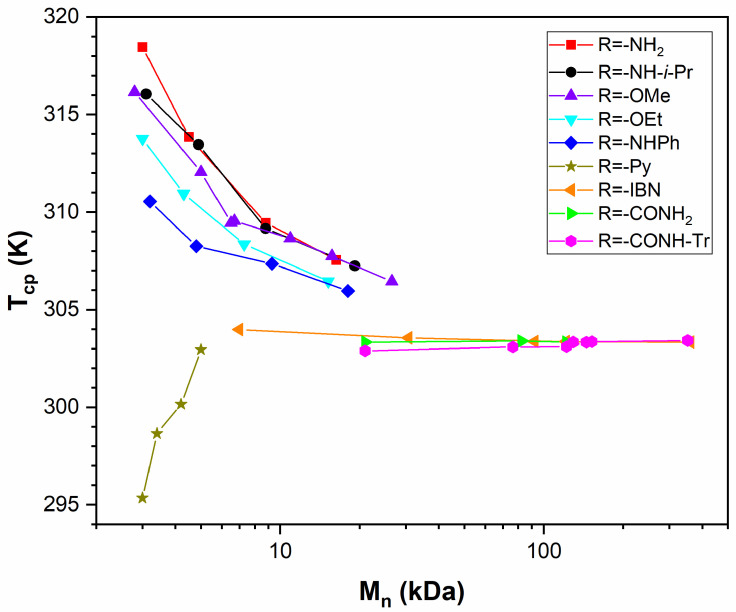
Experimental $T_{cp}$ versus polymer molar mass, $M_{n},$ for narrow-disperse RCOCHCH_3_-(NIPAM)_n_-Cl in aqueous solutions with R = -NH_2_, -NH-*i*-Pr, -OMe, -OEt, and -NHPh $\left(w_{PNIPAM}=0.01\right)$ [13,17] and R = -Py $\left(w_{PNIPAM}=0.002\right)$ [18] and for R-(NIPAM)_n_- in aqueous solutions with R = -IBN, -CONH_2_, and -CONH-Tr $\left(w_{PNIPAM}=0.01\right)$ [29].

In this section, different series of end-functionalized PNIPAMs synthesized by Xia et al. [13,17], Duan et al. [18], and Furyk et al. [29] will be discussed. Xia et al. and Duan et al. prepared, by atom transfer radical polymerization, a set of narrow-disperse PNIPAM samples with the structure RCOCHCH_3_-(NIPAM)_n_-Cl, with R = -NH_2_, -NH-*i*-Pr, -OEt, -OMe, and -NHPh in the range of $2.8\leq M_{n}\leq 26.5$ kDa and R = -Py in the range of $3.0\leq M_{n}\leq 5.0$ kDa, respectively (Scheme 1a). For these PNIPAM samples, $T_{cp}$s were measured for solutions with a mass fraction $w_{PNIPAM}=0.01$ (for the samples of Xia et al.) and $w_{PNIPAM}=0.002$ (for the samples of Duan et al.). In addition, Furyk et al. produced a set of mass-fractionated PNIPAM samples, obtained from polydisperse polymers synthesized by free radical polymerization, with the structure R-(NIPAM)_n_-, with R = -IBN, -CONH-Tr, and -CONH_2_ in the range of $7.0\leq M_{n}\leq 360.0$ kDa (Scheme 1b). The $T_{cp}$s of these samples were also measured with $w_{PNIPAM}=0.01$. The $T_{cp}$ data are summarized in Table S1 and presented in Figure 3 as a function of $M_{n}$. Note that the $M_{n}$ values for the samples of Furyk et al. were calculated from the available $M_{w}$ values obtained from light scattering and the polydispersity index (PDI), defined as $M_{w}/M_{n},$ obtained from SEC data. The PDIs of the Furyk et al. and Xia et al. data are similar.

The measured cloud point data have an experimental uncertainty, which, in most cases, was not mentioned or estimated in the original literature. In some/most cases, a small error on the determined temperature was given. However, the course of $T_{cp}$ with $M_{n}$ in some of the individual data sets makes us anticipate that the accuracy of the experimental data is not as good as hoped for and is not only determined by the accuracy of the temperature readings. For instance, in several cases, the shape of the experimental traces of the turbidity and DSC data that were used to estimate $T_{cp}$s varied significantly, making the determination of $T_{cp}$ not straightforward, certainly in view of the exact thermodynamic definition of the cloud point. For this reason, we assumed that all the experimental $T_{cp}$ data points used in this study have an, also arbitrary chosen, error of 1 K. In reference [25], it was suggested to standardize the commonly used cloud point measurement procedures to be able to compare the experimental data from different labs. This is a useful suggestion, but the problem remains that the commonly used methods cannot guarantee that an accurate estimator for the true thermodynamic cloud point, as defined in Section 2.4, is obtained in all cases.

In Figure 3, all the data obtained by Xia et al. [13,17], Duan et al. [18], and Furyk et al. [29] are shown. According to Xia et al., the hydrophilicity of their R groups varies in the order -NH_2_ > -NH-*i*-Pr ≅ -OMe > -OEt > -NHPh, and all the R groups show a decrease in $T_{cp}$ with increasing molar mass. At a constant molar mass, the most hydrophilic R group, -NH_2_, has the highest $T_{cp}$, whereas when increasing the hydrophobicity of the R group, $T_{cp}$ decreases. The change in $T_{cp}$ becomes smaller with increasing molar mass. The authors conjectured that to see an increase in $T_{cp}$ with molar mass, the hydrophobicity of the R group should be further increased.

The PNIPAMs functionalized with R = -NH-*i*-Pr and R = -OMe have practically identical $T_{cp}$ versus molar mass dependences. The authors reasoned that R = -NH-*i*-Pr is the closest in molecular structure to the PNIPAM middle unit, and the -Cl moiety, present in all the polymers investigated by Xia et al., was kept constant and assumed to be similar to the middle unit, or at least to only have an influence that does not alter the $T_{cp}$ versus $M_{n}$ dependence considered to be characteristic of the homopolymer [17]. Moreover, the molar mass dependences of the $T_{cp}$s of the polymers with R = -NH-*i*-Pr and R = -OMe are practically the same, and both polymers were considered to exhibit the intrinsic homopolymer $T_{cp}-M_{n}$ response.

We rephrase these reasonable arguments of Xia et al. in terms of the FH theory for *α*, *ω*-end-functionalized homopolymers, given by Equations (3)–(6). The polymers functionalized with R_1_ = -NH-*i*-Pr, R_1_ = -OMe, and R_2_ = -Cl studied by Xia et al. are examples of Case 2B, i.e., homopolymers with two end groups, $E_{1}$ and $E_{2},$ with $n_{E_{1}}$ and $n_{E_{2}}=1$ that give very similar results to the original homopolymer FH theory: this happens when $\chi_{OM}≅\chi_{OE_{1}},$ $\chi_{OM}≅\;\chi_{OE_{2}}$ and $\chi_{ME_{1}},\chi_{ME_{2}},\chi_{E_{1}E_{2}}≅0$ or the collective contributions of the end groups cancel, i.e., $\left(-\chi_{OM}\left(\frac{M_{E}}{M_{n}}\right)+\chi_{OE_{1}}\left(\frac{M_{E_{1}}}{M_{n}}\right)+\chi_{OE_{2}}\left(\frac{M_{E_{2}}}{M_{n}}\right)-\chi_{ME_{1}}\left(1-\frac{M_{E}}{M_{n}}\right)\left(\frac{M_{E_{1}}}{M_{n}}\right)-\chi_{ME_{2}}\left(1-\frac{M_{E}}{M_{n}}\right)\left(\frac{M_{E_{2}}}{M_{n}}\right)-\chi_{E_{1}E_{2}}\left(\frac{M_{E_{1}}}{M_{n}}\right)\left(\frac{M_{E_{2}}}{M_{n}}\right)\right)≅0$. Alternatively, all the polymers studied by Xia et al. have -Cl in common, and its effect can be incorporated in the homopolymer reference. Thus, the polymers can be treated as *α*-end-functionalized homopolymers ($n_{E_{1}}=1$ and $n_{E_{2}}=0$). The polymers functionalized with R_1_ = -NH-*i*-Pr and R_1_ = -OMe are then homopolymers with one end group, $E_{1},$ that gives very similar results to the original homopolymer FH theory; this happens when $\chi_{OM}≅\chi_{OE_{1}}$ and $\chi_{ME_{1}}≅0$ or the collective contributions of the end groups cancel, i.e., $\left(-\chi_{OM}\left(\frac{M_{E}}{M_{n}}\right)+\chi_{OE_{1}}\left(\frac{M_{E_{1}}}{M_{n}}\right)-\chi_{ME_{1}}\left(1-\frac{M_{E}}{M_{n}}\right)\left(\frac{M_{E_{1}}}{M_{n}}\right)\right)≅0.$ The qualitative notion “very similar” is now to be interpreted as meaning that within the experimental accuracy, it is impossible to make a distinction in the $\chi \left(T\right)$ function between the end groups and middle units. Consequently, it is impossible to determine the effects of the end groups from the experimental data, and for such experimental data, even at small molar masses where normally the end groups have the largest influence, they can now be used to extract the intrinsic $\chi_{OM}\left(T\right)$.

**Figure 4:**
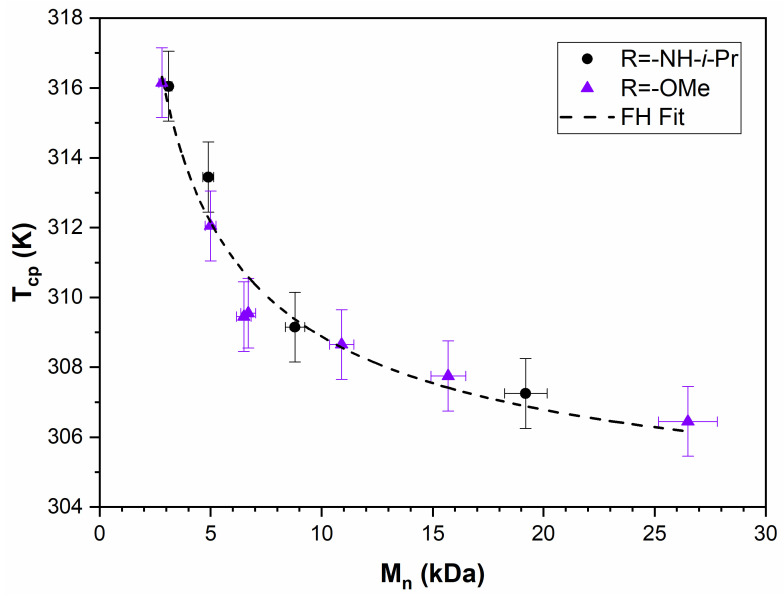
Experimental $T_{cp}$ versus polymer molar mass, $M_{n},$ for PNIPAM homopolymer reference system in water $\left(w_{PNIPAM}=0.01\right)$ [13,17] (symbols) and the FH fit with $\chi_{OM,0}=3.1595\;\mathrm{and}\;\chi_{OM,1}=-805.34$ K (dashed line).

Utilizing the universal prediction according to the FH theory for $\chi_{cp}\left(\phi_{cp},s_{P}\right)$ in Equation (7) together with the $\chi_{IJ}\left(T\right)=\chi_{IJ,0}+\chi_{IJ,1}/T$ behavior, the best fit for $T_{cp}\left(M_{n}\right)$ was obtained with $\chi_{OM,0}=3.1595$ and $\chi_{OM,1}=-805.34$ K, as presented in Figure 4. The $\chi_{OM,0}$ and $\chi_{OM,1}$ values were obtained by employing a parameter estimation program (P.E.P.) [50] with the assumption that the relative error for $M_{n}$ is 5% and the absolute error for $T_{cp}$ is 1 K.

Having now studied the “intrinsic” homopolymer response, we can study systematically the influence of end groups. As discussed in Section 2.5, the effect of the end groups can be separated from that of the middle repeat units. The data of Xia et al. and Duan et al. systematically varied one end group (R = -NH_2_, -NH-*i*-Pr, -OMe, -OEt, -NHPh, and -Py) in the RCOCHCH_3_-(NIPAM)_n_-Cl system. The systems with R = -NH_2_, -OEt, -NHPh, and -Py were chosen to represent a more hydrophilic, slightly less hydrophilic, less hydrophilic, and (very) hydrophobic end group, respectively. These polymers can be theoretically treated to be functionalized at one polymer chain end, and thus Equation (9), with known $\chi_{OM}$, can be applied to study the effect of end groups. The R-(NIPAM)_n_- systems of Furyk et al. (R = -IBN, -CONH_2_, -CONH-Tr) were also treated as polymers with one end group. As the authors did not specify the second end group, we assume it to be fixed, and the effect of this end group is incorporated in $\chi_{OM}$. Note that the linkers (if present) (e.g., COCHCH_3_ and (CH_2_)_2_CCNCH_3_) are included in the definition of the corresponding end groups (see the Supplementary Materials, Table S4, Section R groups versus corresponding end groups in PNIPAM polymers). Accordingly, the $\Delta \chi_{E_{1}}$ values for the PNIPAM polymers with varying end groups were calculated using Equation (9). The standard deviations of these $\Delta \chi_{E_{1}}$ values were determined from the relative error (5%) of $M_{n}$ and the absolute error (1 K) of $T_{cp}$.

**Figure 5:**
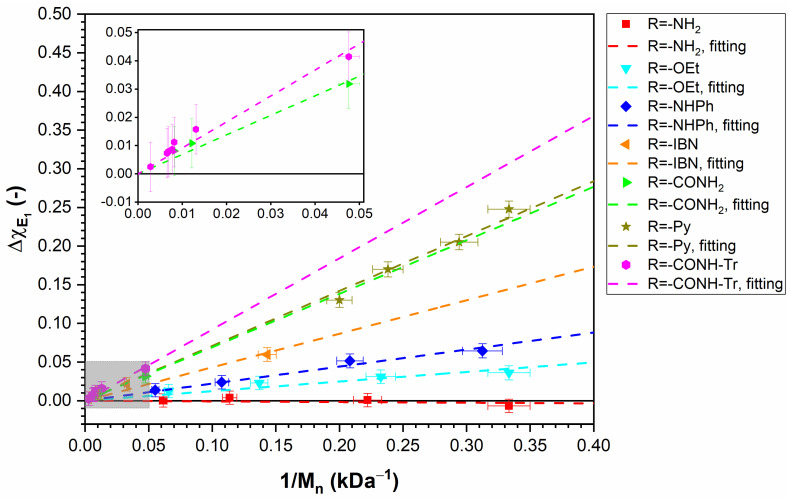
$\Delta \chi_{E_{1}}$ versus the reciprocal of the polymer molar mass, $\frac{1}{M_{n}},$ for PNIPAM polymers with different R groups (symbols) and the FH linear fits of the data using Equation (9) (dashed lines). The black solid line shows the PNIPAM homopolymer reference system. The zoomed-in graph is for the results of Furyk et al.

According to Equation (9), $\Delta \chi_{E_{1}}$ depends on both T (in the form of $\chi_{IJ}=\chi_{IJ,0}+\chi_{IJ,1}/T$) and $M_{n}$. For all experimental data, the temperature range, ΔT, covered is small (ΔT≅11 K at most), and the relative variation, ΔT/〈T〉, is just a few percent. In contrast, the wider range of polymer molar masses ($\Delta M_{n}≅350$ kDa) and their relative variation $\Delta M_{n}/\langle M_{n}\rangle$ is a few 100 percent, thus one to two orders of magnitude larger than ΔT/〈T〉. Therefore, it is safe to assume that the changes in $\Delta \chi_{E_{1}}$ are dominated by $M_{n}$, which is confirmed in the plots of $\Delta \chi_{E_{1}}$ versus $1/M_{n},$ showing a clear correspondence, whereas $\Delta \chi_{E_{1}}$ versus 1/T in practically all cases does not show a clear trend (plots not shown). Therefore, we will focus on the variations in $\Delta \chi_{E_{1}}$ with $1/M_{n},$ as presented in Figure 5. A zoomed-in graph is also added in Figure 5 to clearly show the results of Furyk et al. in the high polymer molar mass range. Evidently, the variations in $\Delta \chi_{E_{1}}$ with $1/M_{n}$ are practically linear, indicating that the *contribution* of the $a_{2}/M_{n}^{2}$ term in Equation (9) is much smaller than the linear term and cannot be determined from the available data with any confidence and must be set effectively equal to 0, i.e., $a_{2}=\chi_{ME_{1}}{\left(M_{E_{1}}\right)}^{2}=0$; thus, $\chi_{ME_{1}}=0$ and $a_{1}=\chi_{OE_{1}}M_{E_{1}}$. The values of $a_{1}$ obtained for the different end groups are summarized in Table 2.

From Figure 5, a clear trend in the molar mass dependence of $\Delta \chi_{E_{1}}$ on the type of end group can be observed. The most hydrophilic R group (R = -NH_2_) has $\Delta \chi_{E_{1}}<0$, whereas the less hydrophilic R groups and the hydrophobic R groups (R = -OEt, -NHPh, -Py, -IBN, -CONH_2_, and -CONH-Tr) have $\Delta \chi_{E_{1}}>0$, as expected. The value $\left|\Delta \chi_{E_{1}}\right|$ is largest at the lowest molar mass and decreases with increasing molar mass, indicating that the effect of the end groups is most significant at the lowest molar mass and becomes less important when the molar mass increases. In addition, the $\Delta \chi_{E_{1}}$ values increase in the order -NH_2_ < -NH-*i*-Pr ≅ -OMe < -OEt < -NHPh < -IBN < -CONH_2_ < -Py < -CONH-Tr, corresponding to the increase in the hydrophobicity of the end groups confirmed by the experimental results. Thus, in the FH theory for *α*, *ω*-end-functionalized homopolymers, the $\Delta \chi_{E_{1}}$ values give a consistent quantification of the influence of the end groups on $T_{cp}$s. Furthermore, the theory also clarifies that the effect of the end groups on $T_{cp}$s is highly nonlinear and related to the interactions of the different units present in the polymer (middle units and end groups) with each other and with the solvent. In the experimental results presented here, it is also shown that the interactions with the solvent are dominant and that the interactions between the different polymer units are sufficiently small, as these interactions would be evident in the case of a quadratic dependence of $\Delta \chi_{E_{1}}$ on $1/M_{n}$.

**Figure 6:**
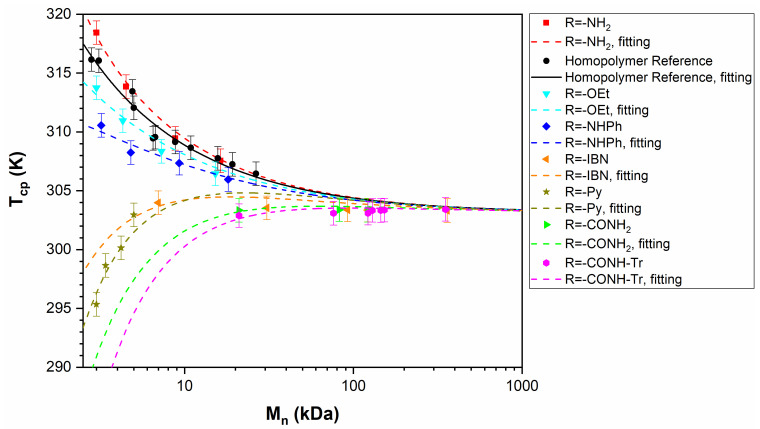
Experimental $T_{cp}$ versus polymer molar mass, $M_{n}$, (symbols) and the FH fits of the data using Equations (6)–(9) (lines) for PNIPAM polymers in water with varying hydrophilicity of the R group: R = -NH_2_, -OEt, and -NHPh and PNIPAM homopolymer reference system (data for R = -NH-*i*-Pr and R = -OMe) [13,17]; and R = -Py [18]; R = -IBN, -CONH_2_, and -CONH-Tr [29]. The standard errors of $M_{n}$ are not plotted to avoid cluttering the figure.

Finally, all the experimental $T_{cp}$ data, together with all the corresponding fitting curves using the coefficients obtained from FH linear fits, are plotted in Figure 6 as a function of $M_{n}$. The FH theory amended for end groups represents the experimental data quite well. A clear trend in the molar mass dependence of $T_{cp}$ on the type of end group can also be observed. In particular, for R = -NH_2_ (more hydrophilic), the end group has $\Delta \chi_{E_{1}}\leq 0$ and results in a steeper decrease in $T_{cp}$ compared to that of the reference or neutral end group ($\Delta \chi_{E_{1}}=0$, the assigned “homopolymer” reference). The end groups with $\Delta \chi_{E_{1}}$ modestly larger than 0 (the less hydrophilic R groups) lead to less steep decreases in the cloud point curves (R = -OEt, -NHPh). For the largest $\Delta \chi_{E_{1}}$ investigated (the most hydrophobic R groups (R = -CONH_2_, -CONH-Tr)), the cloud point curves increase monotonously with $M_{n},$ converging to $T_{cp}^{\infty }$ in the limit $M_{n}\to \;\infty$. For some end groups $(\Delta \chi_{E_{1}}>0$) (intermediate hydrophobic, (R = -IBN)), $T_{cp}$ first increases with $M_{n}$, reaches a maximum, $T_{cp}^{max},$ at an intermediate value of $M_{n},$ and then converges to $T_{cp}^{\infty }$ in the limit $M_{n}\to \;\infty$. Furthermore, all the cloud point curves approach each other asymptotically at sufficiently high molar masses as the effect of the end groups becomes negligible and the behavior of the homopolymer “without end groups” is recovered.

The LCSTs, $T_{cp}$s depicted in Figure 3 and determined by Duan et al. [18] for aqueous solutions of Py-(NIPAM)_n_-Cl polymers $\left(w_{PNIPAM}=0.002\right)$ show that $T_{cp}$ increases with molar mass. The authors investigated the same polymers at the same solution concentrations by static light scattering at temperatures below the reported $T_{cp}$ (the smallest molar mass at 15 °C and the other three molar masses at 20 °C) and determined the *apparent* molar mass of the PNIPAM to be much larger than the molar mass given in Table S1 and determined by NMR and MALDI-TOF. The authors concluded that in the aqueous solutions, aggregates of Py-PNIPAM molecules are present, thereby casting uncertainty about the accuracy and even the meaning of the reported $T_{cp}.$ The determination of the true thermodynamic cloud point, as defined in Section 2.5, starts with the prerequisite that a truly homogenous solution is formed. It is known that under specific conditions, PNIPAM can form, for instance, micelles, mesoglobules, etc., as mentioned in the Introduction. In situations where aggregates, micelles, or other kinds of structures are formed in a homogenous solution before the intended L-L phase separation sets in, the interpretation of what is assumed to be a cloud point according to turbidity measurements or DSC data is not necessarily straightforward, as the interference of these other phenomena should be taken into account or should be prevented (e.g., by working below the critical micelle concentration (cmc) of the system) so that the true thermodynamic $T_{cp}$ defined in Section 2.5 can be applied.

In Figure 3, the different R-PNIPAM samples, with R = -IBN, -CONH_2_, and -CONH-Tr, studied by Furyk et al. are plotted as well [29]. They concluded that in the available molar mass range, the studied R groups have little influence on $T_{cp}$. The effect of the R groups agrees with the order of hydrophilicity observed by Xia et al.; that is, -IBN > -CONH_2_ > -CONH-Tr. The polymers with the most hydrophobic R group (-CONH_2_, -CONH-Tr) have $T_{cp}$ increasing with the molar mass. The $T_{cp}$ results are presented quite well by the FH theory amended for end groups, and for these R-PNIPAMs, the cloud point curves are *predicted* to increase monotonously with $M_{n}$ up to the point where the effect of the end groups can be ignored. More importantly, although in the available molar mass range the absolute magnitude of $\Delta \chi_{E_{1}}$ is very small, the theory predicts that these end groups have large values for the $a_{1}$ parameter, characterizing them to be (very) hydrophobic R groups. For the R group R = -IBN, the estimated $a_{1}$ parameter is smaller than the $a_{1}$ values of R = -CONH_2_ and -CONH-Tr. The FH theory *predicts* that $T_{cp}$ first increases with molar mass, reaches a maximum, and subsequently decreases to the asymptotic value where the effect of end groups can be ignored.

The Furyk et al. and Duan et al.’s light scattering observations for R = -Py and R = -IBN may appear to be contradictory. However, this is not necessarily so; Furyk et al. performed the light scattering experiments in methanol solutions, which are better solvents than water. Furthermore, the effect of end groups becomes less important with increasing molar mass. The molar masses investigated by Furyk et al. are an order of magnitude or larger than the molar masses investigated by Duan et al. Hence, it is very well possible that the results of Furyk et al. are indeed not plagued by aggregate formation, and the $T_{cp}$ results indeed represent the true thermodynamic response. If these authors had prepared much smaller molar masses, they could also have been confronted with aggregates in the $T_{cp}$ experiments.

Qiu et al. reported for the telechelic PNIPAM-*n*Bu system a striking example of invariance in $T_{cp}$ with molar mass [24]. As noted by the authors, the limiting value of $T_{cp}$ at high molar mass is different from values in the literature. The cause of this slight difference is not clear. Therefore, we will not analyze the experimental data in ref. [24] but use our PNIPAM homopolymer reference $\chi_{OM}=3.1595-805.34/T$ to create a hypothetical invariant end-functionalized homopolymer.

This invariance can be predicted by the FH theory and can be reached for both a single and two distinct end groups. The FH theory predicts invariance to occur as a result of the presence of the quadratic 1/M  dependence in Equations (6), (8), or (9). The details of the analysis are given in Supplementary Materials, Section S.2.

### 3.4. PTEGSt

**Scheme 2:**
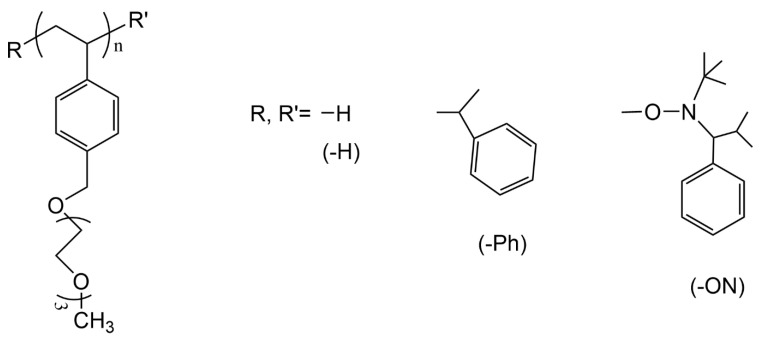
Chemical structure of narrow-disperse R-(TEGSt)_n_-R′ investigated with R, R′ = -H, -Ph, and -ON [20].

**Figure 7:**
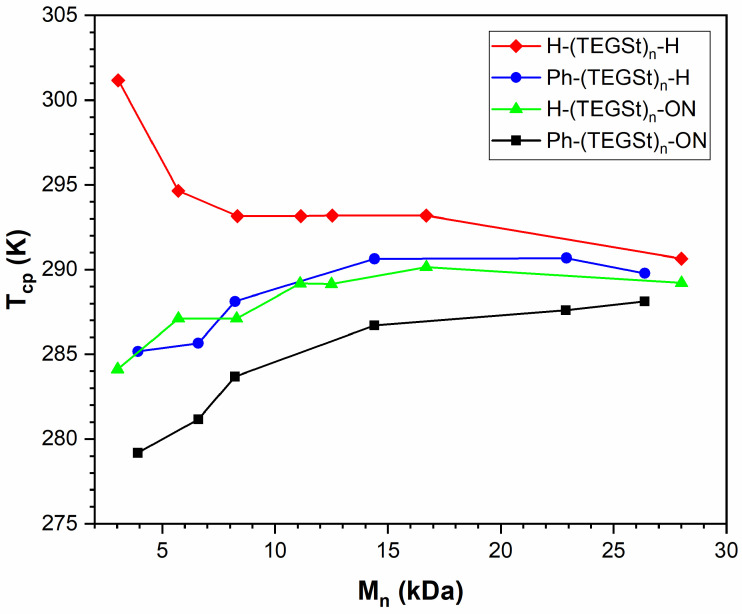
Experimental $T_{cp}$ versus polymer molar mass, $M_{n},$ for PTEGSt polymers with different end groups in aqueous solutions $\left(w_{PTEGSt}=0.01\right)$ [20].

Hua et al. [14] and Jiang and Zhao [20] synthesized, by nitroxide-mediated radical polymerization (NMRP), well-defined *α*, *ω*-end-functionalized PTEGSts carrying phenylethyl (-Ph) and alkoxyamine (-ON) hydrophobic end groups in the range of $3.0\leq M_{n}\leq 28.0$ kDa, PDI < 1.2. In total, four sets of *α*, *ω*-end-functionalized PTEGSts were prepared: H-(TEGSt)_n_-H (homopolymer), Ph-(TEGSt)_n_-H, H-(TEGSt)_n_-ON, and Ph-(TEGSt)_n_-ON, with -H considered to be a neutral end group, making PTEGSt a polymer belonging to Case 2B. The chemical structures of these polymers are illustrated in Scheme 2. Note that for PTEGSt polymers, the end groups used in this work are identical to the corresponding R groups in the original paper. The $T_{cp}$s of all the polymers in water were measured for $w_{PTEGSt}=0.01$ as a function of the molar mass. The experimental $T_{cp}$ and $M_{n}$ data from the original work by Jiang and Zhao were digitized using Origin 2018b [49] and are shown in Figure 7 and Table S2. From Figure 7, the $T_{cp}$s of the homopolymer (H-(TEGSt)_n_-H) decreased with increasing molar mass and was interpreted to be the “true” intrinsic molar mass dependence of $T_{cp}$ in the context of the classic homopolymer FH theory. For polymers with only one end group (either -Ph or -ON), $T_{cp}$s initially increase with molar mass, reach a broad maximum, and then decrease with a further increase in molar mass. However, polymers with two end groups (-Ph and -ON) displayed a monotonous increase with molar mass. The authors interpreted that the effect of the molar mass on the $T_{cp}$ of polymers with one or two end groups was the combined effect of the end groups with the true molar mass influence. It was also mentioned by the authors that—at a similar molar mass—the $T_{cp}$s of Ph-(TEGSt)_n_-H are slightly higher than those of H-(TEGSt)_n_-ON since the -Ph group is less hydrophobic than the -ON group except for the $T_{cp}$ of Ph-(TEGSt)_n_-H at $M_{n}=6.6\;$ kDa, which is lower than any $T_{cp}$ of H-(TEGSt)_n_-ON. This makes us suspect that this data point is an outlier, and it is not considered in the subsequent $\Delta \chi_{E_{1}}$ versus $1/M_{n}$ analysis.

**Figure 8:**
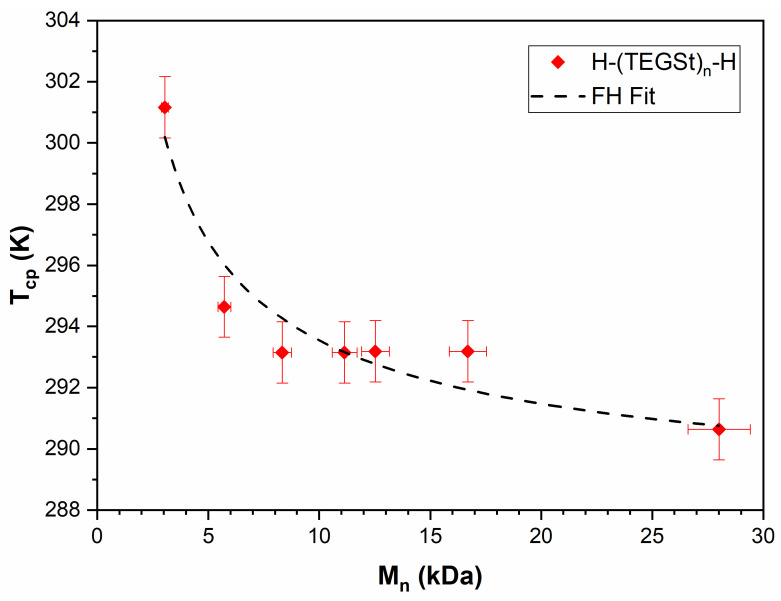
Experimental $T_{cp}$ versus polymer molar mass, $M_{n},$ for H-(TEGSt)_n_-H homopolymer in water $\left(w_{PTEGSt}=0.01\right)$ [20] (symbols) and the FH fit with $\chi_{OM,0}=3.0576$ and $\chi_{OM,1}=-735.44$ K (dashed lines).

The PTEGSt data were analyzed in the same manner as the PNIPAM data in Section 3.3. The FH interaction function of the PTEGSt homopolymer was determined from fitting the experimental $T_{cp}\left(M_{n}\right)$ data to Equation (7), together with $\chi_{OM}\left(T\right)=\chi_{OM,0}+\chi_{OM,1}/T$, resulting in $\chi_{OM,0}=3.0576$ and $\chi_{OM,1}=-735.44$ K. The best fit and experimental data are presented in Figure 8. Fitting was performed using P.E.P. [50] with the assumption that the relative error for $M_{n}$ is 5% and the absolute error for $T_{cp}$ is 1 K, as used for PNIPAM.

We must conclude that the practically constant value of the experimental $T_{cp}$ data in the molar mass range [8–17 kDa] cannot be fitted with the monotonous decrease in $T_{cp}$ with molar mass predicted by the FH theory, as indicated by the dashed line in Figure 8. However, based on a statistical analysis of the agreement between the theoretical fit and the experimental data, the experimental data are still covered by the 0.95 confidence interval, and the statistical parameters t-statistics and *p*-value are still acceptable.

**Figure 9:**
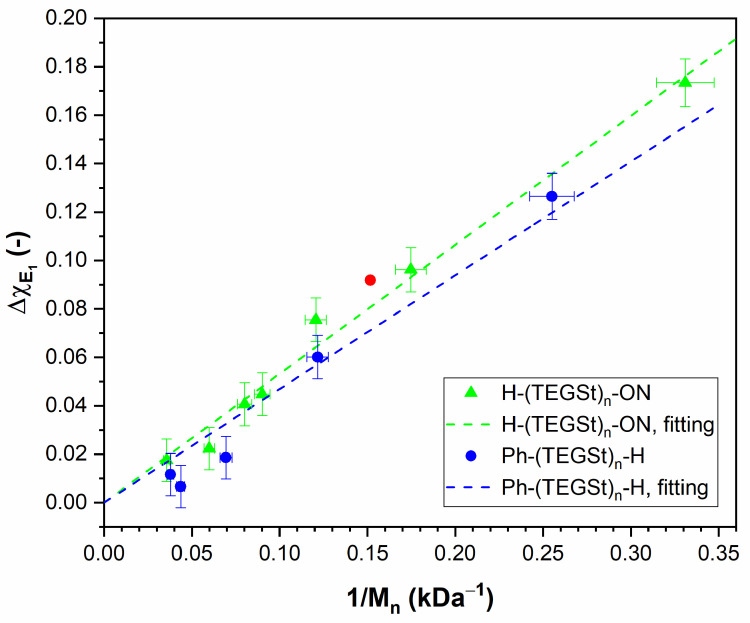
$\Delta \chi_{E_{1}}$ versus the reciprocal of the polymer molar mass, $\frac{1}{M_{n}},$ for Ph-(TEGSt)_n_-H and H-(TEGSt)_n_-ON (symbols) and the FH linear fits of the data using Equation (9) (dashed lines). The excluded data point of Ph-(TEGSt)_n_-H at $M_{n}=6.6\;$ kDa is shown in red.

Having now studied the available FH interaction parameters corresponding to the “intrinsic” homopolymer response, the effect of the end groups can again be separated from that of the middle repeat units using Equation (9). We apply the same approach and approximations as argued in the case of PINIPAM, i.e., although $\Delta \chi_{E_{1}}$ depends on both T and $M_{n}$, the relative variation in $M_{n}$ is at least more than an order of magnitude larger than the relative variation in T. Therefore, the changes in $\Delta \chi_{E_{1}}$ are dominated by $M_{n}$, which is confirmed in the plots of $\Delta \chi_{E_{1}}$ versus $1/M_{n},$ showing a clear correlation, as presented in Figure 9. Evidently, the variations in $\Delta \chi_{E_{1}}$ with $1/M_{n}$ are practically linear, indicating that the *contribution* of the $a_{2}/M_{n}^{2}$ term in Equation (9) is much smaller than that of the linear term, and the quadratic term must be set effectively equal to 0, i.e., $a_{2}\equiv \chi_{ME_{1}}{\left(M_{E_{1}}\right)}^{2}=0$; thus, $\chi_{ME_{1}}=0$ and $a_{1}=\chi_{OE_{1}}M_{E_{1}}$. The values of $a_{1}$ obtained for the different end groups are summarized in Table 3.

As can be seen in Figure 9, the linear fits of $\Delta \chi_{E_{1}}$ versus $1/M_{n}$ for the PTEGSt polymers containing a -Ph or -ON end group were applied, and the results show reasonable agreement between the data points and the fitted lines. Jiang and Zhao [20] indicated that the -ON end group is slightly more hydrophobic than the -Ph end group, and this is confirmed by the larger $\Delta \chi_{E_{1}}$ values for the -ON end group than for the -Ph end group, as can be seen in Figure 9, and thus, there is also a correspondingly larger value for the parameter $a_{1}$ for the -ON end group than for the -Ph end group.

With the $a_{1}$ coefficients for the -Ph and -ON end groups available, we can now predict (approximately) the $T_{cp}$ versus $M_{n}$ dependence of the Ph-(TEGSt)_n_-ON polymer. According to the theory (see Equation (9)) and taking into account that experimentally $\chi_{ME_{1}}=0\;$ and $\chi_{ME_{2}}=0$ in view of the experimentally observed linear dependence of $\Delta \chi_{E_{i}}$ versus $1/M_{n}$, we can (safely) assume that it is most likely that $\chi_{E_{1}E_{2}}$ will also be small, and we set $\chi_{E_{1}E_{2}}=0$. With these assumptions, Equation (8) becomes

(11) $$\Delta \chi_{E}=\frac{a_{1}}{M_{n}}$$

with $a_{1}=\chi_{OE_{1}}M_{E_{1}}+\chi_{OE_{2}}M_{E_{2}}=a_{1,E_{1}}+a_{1,E_{2}}$.

Hence, the $T_{cp}$ versus $M_{n}$ dependence of the -ON, -Ph-end-functionalized PTEGSt can be predicted from the data of the mono-functionalized PTEGSt, using for the bi-functionalized polymer in $\Delta \chi_{E}=\left(a_{1,E_{1}}+a_{1,E_{2}}\right)/M_{n}=\left(a_{1}\right)/M_{n}$ together with Equation (7). The predicted value of $a_{1}$ of the *α*, *ω*-end-functionalized PTEGSt is also given in Table 3 (last row).

**Figure 10:**
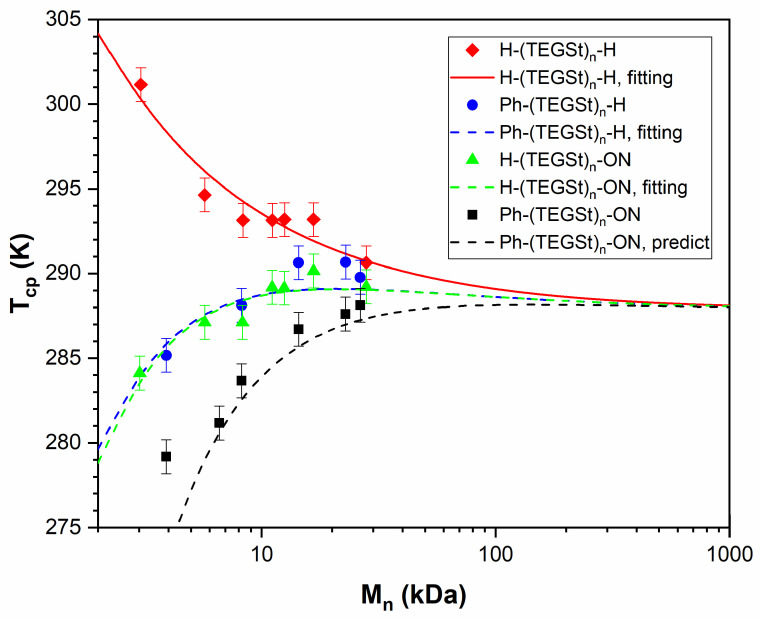
Experimental $T_{cp}$ versus polymer molar mass, $M_{n},$ for PTEGSt polymers in water with varying hydrophilicity of the end group(s) [20] (symbols) and the FH fits/predictions of the data using Equations (6)–(9) (lines). The standard errors of $M_{n}$ are not plotted to avoid cluttering the figure.

In Figure 10, all the experimental $T_{cp}$ data for the PTEGSt polymers are plotted as a function of $M_{n}$, together with the FH-calculated $T_{cp}$ versus $M_{n}$ curves. The FH curve of Ph-(TEGSt)_n_-H stays slightly above that of H-(TEGSt)_n_-ON. This result is consistent with the experimental observation of Jiang and Zhao. The experimental $T_{cp}$ versus $M_{n}$ data of the Ph-(TEGSt)_n_-ON polymer and the FH-predicted $T_{cp}$ versus $M_{n}$ curve are in quite good agreement, indicating the effectiveness of the FH theory amended for end groups. A clear trend in the molar mass dependence of $T_{cp}$ on the type of end group can be observed. In particular, the PTEGSt homopolymer shows a monotonous decrease in $T_{cp}$ with $M_{n}$, converging to $T_{cp}^{\infty }$ in the limit $M_{n}\to \;\infty$. PTEGSt containing one hydrophobic end group (-ON or -Ph) initially exhibits an increase in $T_{cp}$ versus $M_{n}$, reaching a weak maximum and subsequently slowly reaching the asymptotic value where the effect of end groups becomes negligible. The predicted trend for PTEGSt containing two hydrophobic end groups (-ON and -Ph) displays a monotonous increase in $T_{cp}$ with $M_{n},$ converging to $T_{cp}^{\infty }$ in the limit $M_{n}\to \;\infty$ without passing a maximum, which is in agreement with the experimental data.

### 3.5. PHTrEGSt

**Scheme 3:**
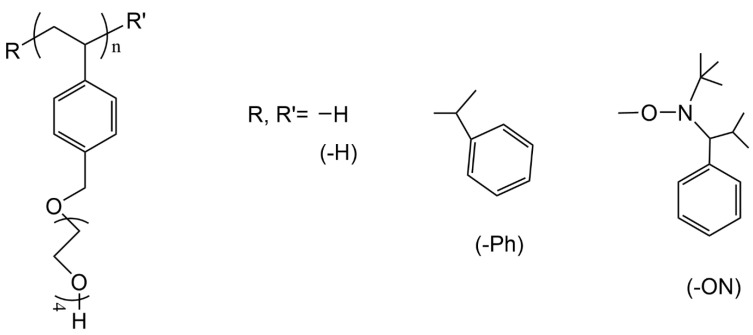
Chemical structure of narrow-disperse R-(HTrEGSt)_n_-R′ investigated with R, R′ = -H, -Ph, and -ON [20].

**Figure 11:**
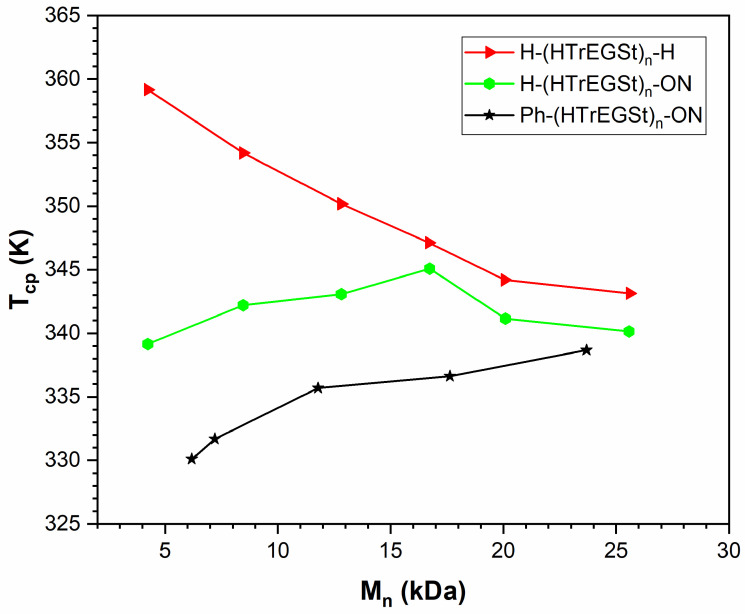
Experimental $T_{cp}$ versus polymer molar mass, $M_{n},$ for PHTrEGSt polymers with different end groups in aqueous solutions $\left(w_{PHTrEGSt}=0.01\right)$ [20].

Three sets of well-defined *α*, *ω*-end-functionalized PHTrEGSt polymers bearing -Ph and -ON hydrophobic end groups in the range of $4.2\leq M_{n}\leq 25.6$ kDa and PDI < 1.2 were also prepared by Hua et al. [14] and Jiang and Zhao [20] by NMRP: H-(HTrEGSt)_n_-H (homopolymer), H-(HTrEGSt)_n_-ON, and Ph-(HTrEGSt)_n_-ON. Their chemical structures are illustrated in Scheme 3. Note that the end groups investigated in this section are identical to the corresponding R groups in the original paper. The $T_{cp}$s of these polymers in water were determined for $w_{PHTrEGSt}=0.01$ as a function of molar mass. The experimental $T_{cp}$ and $M_{n}$ data from the original paper were also digitized using Origin 2018b [49] and are presented in Figure 11 and Table S3.

In Figure 11, similar variations in $T_{cp}$ versus $M_{n}$ for the PHTrEGSt polymers can also be observed as for the aforementioned PTEGSt polymers, i.e., a decrease in $T_{cp}$ with $M_{n}$ as the “true” intrinsic molar mass dependence of $T_{cp}$ in the context of the classic homopolymer FH theory observed for PHTrEGSt homopolymers, an initial increase in $T_{cp}$ with molar mass, reaching a broad maximum, followed by a slight decrease when further increasing the molar mass for the PHTrEGSt polymers containing only one end group (-ON), and a monotonous increase in $T_{cp}$ with molar mass for the PHTrEGSt polymers with two end groups (-Ph and -ON). The authors also interpreted that the effect of the molar mass on the $T_{cp}$ of the PHTrEGSt polymers containing one or two end groups was the combined effect of the end groups with the true molar mass influence. This conclusion can be quantitatively reinterpreted by using the FH expression for the excess Gibbs energy of mixing for an *α*, *ω*-end-functionalized monodisperse homopolymer in solution, as given in Equation (3), with χ shown in Equation (6).

**Figure 12:**
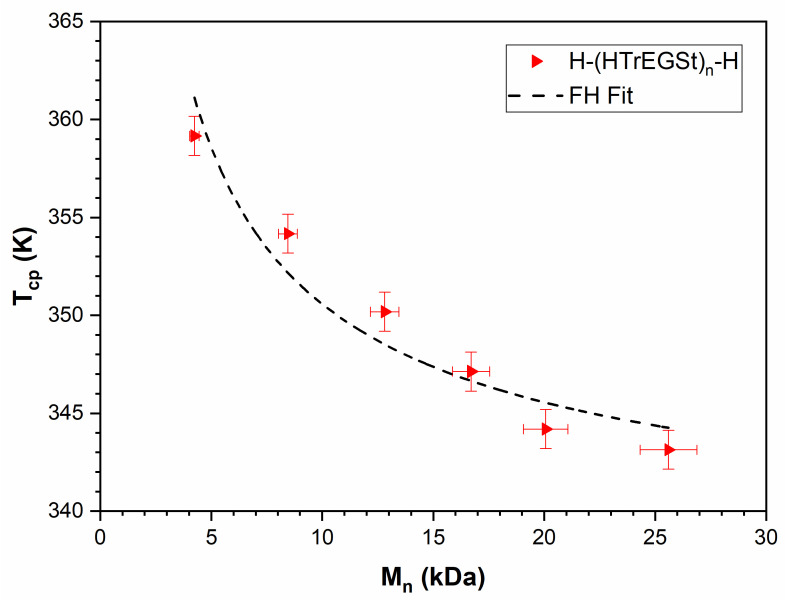
Experimental $T_{cp}$ versus polymer molar mass, $M_{n},$ for H-(HTrEGSt)_n_-H homopolymer in water $\left(w_{PHTrEGSt}=0.01\right)$ [20] (symbols) and the FH fit with $\chi_{OM,0}=1.7782$ and $\chi_{OM,1}=-429.81$ K (dashed lines).

The PHTrEGSt data were also analyzed in the same manner as the PNIPAM data and the PTEGSt data in Section 3.3 and Section 3.4. The FH interaction function of the PHTrEGSt homopolymer was determined from fitting the experimental $T_{cp}\left(M_{n}\right)$ data to Equation (7), together with $\chi_{OM}\left(T\right)=\chi_{OM,0}+\chi_{OM,1}/T$, resulting in $\chi_{OM,0}=1.7782$ and $\chi_{OM,1}=-429.81$ K. The best fit and experimental data are presented in Figure 12. Fitting was performed using P.E.P. [50] with the assumption that the relative error for $M_{n}$ is 5% and the absolute error for $T_{cp}$ is 1 K, as used for PNIPAM and PTEGSt.

**Figure 13:**
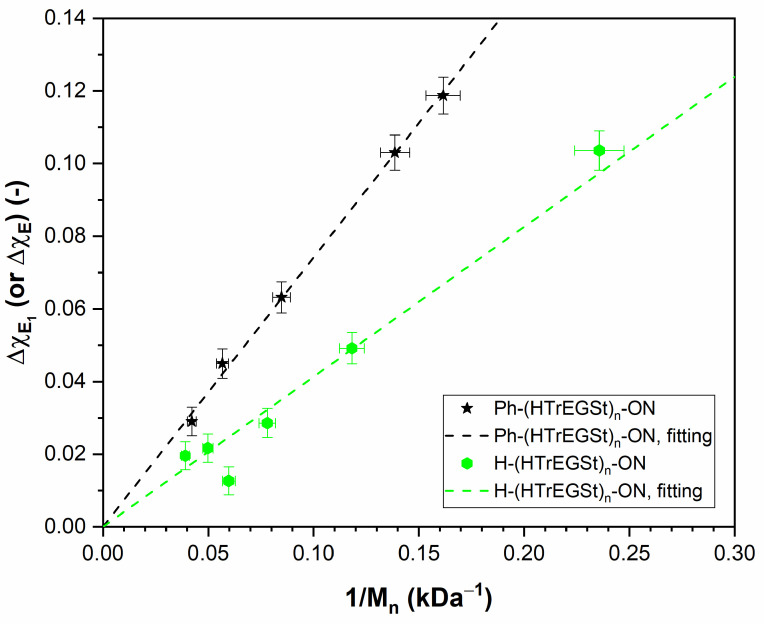
$\Delta \chi_{E_{1}}$ (or $\Delta \chi_{E}$) versus the reciprocal of the polymer molar mass, $\frac{1}{M_{n}},$ for H-(HTrEGSt)_n_-ON and Ph-(HTrEGSt)_n_-ON (symbols) and the FH linear fits of the data using Equations (9) and (11) (dashed lines).

Having now studied the available FH interaction parameters corresponding to the “intrinsic” homopolymer response, the effect of the end groups can be extracted using Equations (8) and (9). Note that, in the case of PHTrEGSt polymers, only the $T_{cp}(M_{n})$ data for PHTrEGSt containing one end group (-ON) and PHTrEGSt containing two end groups (-ON and -Ph) are available; the $T_{cp}(M_{n})$ data for PHTrEGSt containing one end group (-Ph) are unavailable. Applying the same approach and approximations as argued in the cases of PINIPAM and PTEGSt, i.e., although $\Delta \chi_{E_{1}}$(or $\Delta \chi_{E}$) depends on both T (in the form of $\chi_{IJ}\left(T\right)=\chi_{IJ,0}+\chi_{IJ,1}/T$) and $M_{n}$, the relative variation in $M_{n}$ is at least more than an order of magnitude larger than the relative variation in T. Therefore, the changes in $\Delta \chi_{E_{1}}$(or $\Delta \chi_{E}$) are dominated by $M_{n}$, which is confirmed in the plots of $\Delta \chi_{E_{1}}\;$(or $\Delta \chi_{E}$) versus $1/M_{n},$ showing a clear correspondence, as presented in Figure 13. Evidently, the variations in $\Delta \chi_{E_{1}}$ (or $\Delta \chi_{E}$) with $1/M_{n}$ are practically linear, indicating that the *contribution* of the quadratic term in Equations (8) and (9) is much smaller than that of the linear term, and the quadratic term must be set effectively equal to 0, i.e., $\chi_{ME_{1}}{\left(M_{E_{1}}\right)}^{2}$, $\chi_{ME_{2}}{\left(M_{E_{2}}\right)}^{2}$, and $\chi_{E_{1}E_{2}}M_{E_{1}}M_{E_{2}}=0$; thus, $\chi_{ME_{1}}$, $\chi_{ME_{2}}$, $\chi_{E_{1}E_{2}}=0$ and $a_{1}=\chi_{OE_{1}}M_{E_{1}}$ (for PHTrEGSt with -ON end group) or $a_{1}=\chi_{OE_{1}}M_{E_{1}}+\chi_{OE_{2}}M_{E_{2}}=a_{1,E_{1}}+a_{1,E_{2}}$ (for PHTrEGSt with -ON and -Ph end groups), as shown in Equation (11). The values of $a_{1}\;$ obtained for the different end groups are summarized in Table 4. As can be seen in Figure 13, the linear fits of $\Delta \chi_{E_{1}}$ (or $\Delta \chi_{E}$) versus $1/M_{n}$ for PHTrEGSt polymers containing one end group (-ON) or two end groups (-Ph and -ON) were applied, and the results show reasonable agreement between the data points and the fitted lines.

Although the $T_{cp}$ versus $M_{n}$ data for PHTrEGSt containing a -Ph end group are not available, according to Equation (11), the effects of end groups are additive, and thus, the $T_{cp}$ versus $M_{n}$ dependence of the PHTrEGSt containing an -Ph end group can be predicted from the available coefficients, $a_{1},$ for PHTrEGSt containing an -ON end group and PHTrEGSt containing -Ph and -ON end groups in $\Delta \chi_{E}=\left(a_{1,E_{1}}+a_{1,E_{2}}\right)/M_{n}=a_{1}/M_{n}$ together with Equation (7). The predicted value of $a_{1}$ for the Ph-functionalized PHTrEGSt is also given in Table 4 (last row).

**Figure 14:**
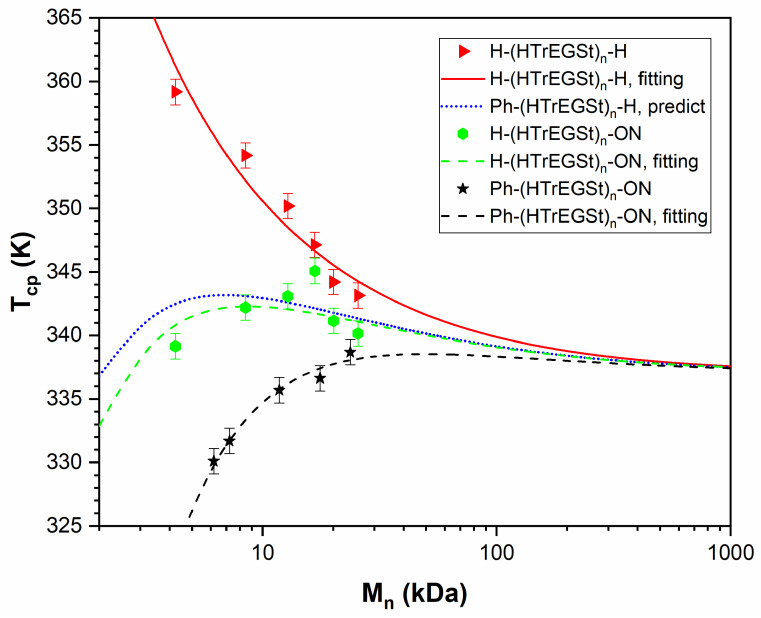
Experimental $T_{cp}$ versus polymer molar mass, $M_{n},$ for PHTrEGSt polymers in water with varying hydrophilicity of the end group(s) [20] (symbols) and the FH fits/predictions of the data using Equations (6)–(9) (lines). The standard errors of $M_{n}$ are not plotted to avoid cluttering the figure.

In Figure 14, all the experimental $T_{cp}$ data for the PHTrEGSt polymers are plotted as a function of $M_{n}$, together with the FH-calculated $T_{cp}$ versus $M_{n}$ curves. The predicted $T_{cp}\left(M_{n}\right)$ curve for -Ph-containing PHTrEGSt is also presented, and the result is consistent with the experimental observations that the $T_{cp}$ of Ph-(HTrEGSt)_n_-H polymers in water is slightly higher than the $T_{cp}$ of H-(HTrEGSt)_n_-ON polymers in water at low polymer molar masses. Also, in Figure 14, a clear trend in the molar mass dependence of $T_{cp}$ on the type of end group can be observed. Particularly, the PHTrEGSt homopolymer shows a monotonous decrease in $T_{cp}$ with $M_{n},$ converging to $T_{cp}^{\infty }$ in the limit $M_{n}\to \;\infty$. PHTrEGSt containing one hydrophobic end group (-ON or -Ph) exhibits an initial increase in $T_{cp}$ versus $M_{n}$, reaching a clear maximum and subsequently slowly reaching the asymptotic value where the effect of end groups becomes insignificant. An initial increase in $T_{cp}$ versus $M_{n}$ with a broad maximum and then converging to $T_{cp}^{\infty }$ in the limit $M_{n}\to \;\infty$ is also shown for PHTrEGSt bearing two hydrophobic end groups (-ON and -Ph). In contrast, PTEGSt containing two hydrophobic end groups (-ON and -Ph) displays a monotonous increase in $T_{cp}$ versus $M_{n}$ but without reaching a maximum (see Section 3.4).

## 4. Discussion

### 4.1. Summary of the Theoretical Results of the FH Theory and Comparison with Other Theories

The conventional FH homopolymer theory was amended for a linear homopolymer with end groups based on the mapping of the FH theory for statistical terpolymers on *α*, *ω*-end-functionalized homopolymers. According to the statistical terpolymer FH theory, the contribution of the excess entropy of the mixing of the statistical terpolymer and the solvent is identical to that of a homopolymer with the same number of segments. In fact, the FH theory is a zero-order theory (in the definition of the lattice cluster theory (LCT) of Freed and coworkers [51]), which predicts that the excess entropy of the mixing of an end-functionalized homopolymer is identical to that of a homopolymer containing the same number of segments on the lattice, i.e., treating the end units to be indistinguishable from the middle units, as discussed in the conventional FH theory of homopolymer solutions (details in Appendix A.1). Information on the terpolymer is only present in the *effective* FH interaction parameter, which is a function of all the FH interaction parameters, $\chi_{IJ}$, representing the ter-monomers, the solvent, and the overall *intramolecular* chemical composition of the terpolymer.

The mapping of the terpolymer theory to an end-functionalized homopolymer in the presence of end groups results in the FH interaction parameter of an end-functionalized polymer in a solution becoming an FH interaction function depending on the molar mass of the end-functionalized homopolymer and on the different FH interaction parameters in the system ($\chi_{OM},\;\chi_{OE_{1}},\;\chi_{ME_{1}},\;\chi_{OE_{2}},\;\chi_{ME_{2}},\;\chi_{E_{1}E_{2}}$), according to Equation (6) (details in Appendix A.1). This could be seen as a drawback of the theoretical approach, but it is not. It is a general principle that the introduction of additional chemical units leads to additional interaction parameters, and to obtain a molecular understanding of the properties of molecules with richer chemical details, the introduction of more information about these details is inevitable.

The FH predictions of $\chi_{cp}\left(M,\;\phi_{cp}\right)$ and the cloud point temperatures, $T_{cp}\left(M,\;\phi_{cp}\right),$ depend in a nonlinear fashion on the molar mass at a given $\phi_{cp}$ (see, e.g., Figure 1 and Figure 2). The “intrinsic” behavior of a homopolymer (without end groups) depends only on $\chi_{OM}$ and can be experimentally observed, as discussed in the different cases identified in Section 2.3.

According to the FH theory, the influence of end groups has been disclosed to be algebraically dependent on 1/M and should vary according to $\Delta \chi_{E}\left(M,\;\phi_{cp}\right)=a_{1}/M+a_{2}/M^{2}$, with a dependence of the parameters $a_{1}$ and $a_{2}$ on the FH interaction parameters defined by Equation (8) or (9) for an *α*, *ω*-end-functionalized and *α*-end-functionalized homopolymer, respectively.

For the simplest conceivable polymer with a single end segment that only differs in its value for $\chi_{OE_{1},1}$ compared to $\chi_{OM,1}$ (i.e., differing only in its excess enthalpy of mixing), the theoretically possible variations in $T_{cp}$ with the polymer chain length are shown in Figure 2 and are in agreement with the experimental behavior of $T_{cp}$ versus M. Accordingly, a neutral end group ($\chi_{OE_{1},1}≅\chi_{OM,1}$) gives a cloud point curve that is the same as for a homopolymer without an end group, i.e., the “intrinsic” behavior. A more hydrophilic end group ($\chi_{OE_{1},1}<\chi_{OM,1}$) yields a steeper cloud point curve. A less hydrophilic/intermediate hydrophobic end group ($\chi_{OE_{1},1}>\chi_{OM,1}$) results either in a less steep cloud point curve than that of a neutral end group or a cloud point curve which initially increases, reaches a maximum, and subsequently slowly reaches the asymptotic value where the effect of the end groups can finally be ignored. For a very hydrophobic end group ($\chi_{OE_{1},1}\gg \chi_{OM,1}$), the cloud point curve asymptotically increases, reaching the infinite molar mass result (the end groups have no effect anymore) without passing through a maximum. Finally, all the cloud point curves converge to the same value, $T_{cp}^{\infty }\left(\phi_{cp}\right),$ at a sufficiently high polymer molar mass. From this first analysis, it is already clear that it is essential to explicitly account for the effects of the end groups in a theory that aims to explain the effect of end groups on the $T_{cp}\;$ of an end-functionalized homopolymer. Any theory discussing the effects of end groups can take advantage of the general observations and conclusions made in this manuscript on the effects of end groups.

### 4.2. The Theoretical Results of the FH Theory in Comparison with Experiments

As explained in the discussion of the theoretical predictions, given $\chi_{OM},$ the influence of the end groups varies according to the FH theory as $\Delta \chi_{E}\left(M,\;\phi_{cp}\right)=a_{1}/M+a_{2}/M^{2}$ (Equation (8) or (9)). For all the experimental systems investigated, it was found that Equation (8) or (9) is indeed obeyed with $a_{2}=0$, meaning that the $\Delta \chi_{E}$ of the end-functionalized polymer in solution is dominated by $\chi_{OE_{1}}$ and $\chi_{OE_{2}}$, i.e., the interactions with the solvent, and that, within the experimental accuracy, the interactions $\chi_{ME_{1}},\chi_{ME_{2},}$ and $\chi_{E_{1}E_{2}}$ are much smaller in magnitude. This is quite reasonable as $\chi_{OM},\chi_{OE_{1}},$ and $\chi_{OE_{2},}$ representing the interactions of the organic moieties of the middle units and end groups in a polymer with water, are expected to be much larger than $\chi_{ME_{1}},\chi_{ME_{2}}$ and $\chi_{E_{1}E_{2}}$, i.e., the interactions between organic moieties. Nevertheless, if $\chi_{ME_{1}},\chi_{ME_{2},}$ or $\chi_{E_{1}E_{2}}$ became sufficiently large, they could have an influence which would manifest in a quadratic (curved) variation in $\Delta \chi_{E}\;$ with 1/M.

Furthermore, despite the nonlinear behavior of the cloud point temperatures with molar mass, the predicted algebraic dependence of $\Delta \chi_{E}$ is also confirmed by the $\Delta \chi_{E}$ derived from the experimental cloud point data. The values of the $a_{1}$ parameters of the set of end groups studied for a particular end-functionalized homopolymer in aqueous solution vary in agreement with the qualitative criterium of hydrophilicity/hydrophobicity used in the literature of experimental data [17,18,20,29].

In many cases, the experimental cloud point data vary with molar mass at least qualitatively and often semi-quantitatively, in agreement with the nonlinear variation predicted by the theory. In particular, the theory predicts a steeper decrease in the cloud point curve observed for (very) hydrophilic end groups, an “intrinsic” decrease in the cloud point curve for neutral end groups, a less steep decrease in the cloud point curve for less hydrophilic end groups, a cloud point curve which initially increases, reaches a maximum, and then decreases to the asymptotic value where the effect of end groups becomes negligible observed for (intermediate) hydrophobic end groups, or a monotonous increase in the cloud point curve up to the point that the effect of end groups can be ignored obtained for (very) hydrophobic end groups. However, it should be noted that for some experimental systems, the predicted FH curves deviate systematically from the experimental data. For these systems, the number of data points is limited, and the experimental $T_{cp}\;$ versus M dependence is difficult to explain with the simple FH theory used in this work. Despite these deviations, the statistical analysis of the theoretical predictions shows that even in these cases, only a limited number of experimental data points are not covered by the 0.95 confidence intervals, and the model parameters still have acceptable values in terms of t-statistics and P-values. As mentioned before, the experimental data are the result of turbidity measurements that do not present the true thermodynamic cloud point (see Section 2.1), or other phenomena are at play for these investigated polymers that are not accounted for by the simple FH theory for end-functionalized homopolymers. Nevertheless, the observed and predicted $\Delta \chi_{E}$ versus 1/M curves are in agreement with the theory, and the qualitative concept of the order of hydrophobicity/hydrophilicity is also obeyed by the predicted $\Delta \chi_{E}$ versus 1/M and $a_{1}=\chi_{OE_{1}}M_{E_{1}}$ values.

### 4.3. Summary of the Theoretical Improvements to the FH Theory

The FH theory for end-functionalized homopolymers in solutions used in the work was obtained by mapping the theory of statistical copolymers in solutions to end-functionalized polymers in solutions, and both theories thus have the same approximations. It is assumed that the end and middle segments are also statistically distributed in the chain, just as is the case for a statistical copolymer. Evidently, in reality, the end units are always at the ends of the chain and never in the middle. This approximation might be essential due to the fact that the intramolecular composition of a copolymer/end-functionalized homopolymer only appears in the *effective* FH interaction parameter in the excess energy of mixing and is absent in the excess entropy of mixing, only depending on the overall solution concentration, i.e., the polymer volume fraction. Therefore, in this section, we will consider a number of theoretical improvements that can be made to the FH theory and evaluate their influence on the (general) predictions made by the FH theory used in this work. Here, we only give a summary of the main conclusions, and the details of these theoretical considerations are presented in the Appendices.

The FH theory is probably the best known approximation of the exact results of a lattice model, although more accurate theoretical approximations are available, such as the expressions for the excess Gibbs energy of mixing given by Huggins [32,33], Orr [52], Guggenheim [53,54,55], and Miller [56,57] and, more recently, the Lattice Cluster Theory (LCT) introduced by Freed and coworkers [51,58,59,60,61,62,63,64,65,66,67,68]. For the simplest FH homopolymer, the LCT is written as a double expansion of the (dimensionless) interaction energy, x≡βΔϵ, and the inverse of the lattice coordination number, $z^{-1}$ [51]. Madden et al. demonstrated that in the framework of the LCT, the FH theory is a first-order perturbation theory in x with respect to an athermal reference system, taking only the zero-order terms in the $z^{-1}$ expansion. The LCT gives an exact theoretical framework for lattice models, facilitating the systematic construction of improved approximations; however, higher-order approximations become increasingly cumbersome to evaluate (see Appendix A.2.2).

The closed theoretical expressions, including the Guggenheim nonrandom mixing theory and the Huggins theoretical approximation, on the other hand, are quite successful improvements, giving closed expressions without the drawbacks of expansions, but they are not amenable to successive improvements.

Huggins obtained in his derivation of what we now call the FH theory a more accurate approximation for the excess entropy of mixing [32,33] (see Appendix A.2.4). Huggins took into account the connectivity of the segments (middle segments always have at least two nearest-neighbor lattice positions occupied by the two neighboring segments in the chain, whereas for end segments, only one nearest-neighbor lattice site is taken by the nearest-neighboring segment of the end segment), and the number of available configurations on the lattice is smaller than assumed in the FH theory, leading to a reduction in the excess entropy of mixing. Accounting for the chain connectivity, the excess energy of mixing in Equation (6) acquires an extra concentration dependence, i.e., $\chi \phi_{O}\phi_{P}\frac{\left(1-\Gamma_{P}\right)}{\left(1-\Gamma_{P}\phi_{P}\right)}$, with $\Gamma_{P}=\gamma \left(1-1/s_{P}\right),\;\gamma =2z^{-1}$ (details Appendix A.2.7).

The theory developed by Guggenheim considers the effects of nonrandom mixing due to differences in the nearest-neighbor potential energies. The effects of nonrandom mixing are expressed in a so-called quasi-chemical equation, and in the present case of a simple FH homopolymer in a solution, the excess Helmholtz energy of mixing can still be presented in closed form [53,54,55] (details Appendix A.2.5). Freed and coworkers reported that the quasi-chemical theory of Guggenheim gives very good agreement with the simulation data [51,58]. For not too large interaction energies, the Guggenheim theory reduces to the Huggins approximation; hence, the Huggins theory is in this case also in very good agreement with these simulation data [51,58,69,70].

Although the closed expressions produced by Guggenheim and Huggins are not easily amenable to systematic improvements, when the interaction energy is not too large, the Huggins expression is a particularly good approximate theory that explicitly considers the distinctions between middle and end segments.

A closer look at the theoretical expression devised by Huggins shows that Huggins’ excess entropy of mixing can be presented almost quantitatively as the sum of the FH excess entropy of mixing plus $\alpha_{m}\;\phi_{P}\left(1-\phi_{P}\right)$, where $\alpha_{m}$ is a theoretical parameter that is a function of the lattice coordination number, z (details are given in Appendix A.2.7). The term $\alpha_{m}\;\phi_{P}\left(1-\phi_{P}\right)$ has exactly the form $\chi_{0}\phi_{P}\left(1-\phi_{P}\right)$. Therefore, the Huggins theory also gives a molecular justification for $\chi_{0}$ in Equation (5), and in the fitting of the parameters in the FH theory for end-functionalized homopolymers, the theoretical Huggins correction will also effectively be taken care of by $\chi_{0}$ (details Appendix A.2.7).

For the excess energy of mixing, the effect of the connectivity rescales the bare value of the FH $\chi_{1}$ to $\chi_{1}\left(1-\Gamma_{P}\right),$ and this effect is automatically taken care of in the fitting of the FH theory with the experimental results. The additional concentration dependence $1/\left(1-\Gamma_{P}\phi_{P}\right)$ is not easily accounted for in the simple FH theory, and it also does not change the predictions of the theory in a qualitative manner, as explained in the next paragraph.

The Huggins theory is easily extended to copolymers and end-functionalized homopolymers in solutions in the same manner as was carried out for the simplest homopolymer in the FH theory (details are given in Appendix A.3). The key point is that in the Huggins theory, the intramolecular composition of the statistical copolymer is also only exposed in the *effective* FH interaction parameter in the excess energy of mixing and not in the excess entropy of mixing, which again only depends on the overall polymer concentration of the polymer solution. Hence, the Huggins excess Helmholtz energy of mixing has exactly the same separation between the intramolecular composition of the terpolymer and the overall polymer concentration of the polymer solution in, respectively, the excess energy of mixing and the excess entropy of mixing as observed in the FH theory for the statistical copolymers and end-functionalized homopolymers discussed in this work (details Appendix A.3.1). It must be noted that the Huggins theory takes the effect of the end segments explicitly into account, and it manages this with gratifying success.

Also, in this case, the intramolecular information is limited to the interaction energy (*effective* χ parameter), and in terms of entropy, the copolymer composition/end functionalization does not appear just as it does in the case of the FH approximation. Although the Huggins theory gives a theoretical improvement, the theoretical improvement is partially considered in the FH approximation; for the entropy, this is reflected in the $\chi_{0}$ parameters. For the interaction energy, the $\chi_{1}$ parameter is renormalized. The additional concentration dependence in the interaction energy is not accounted for.

The inclusion of the theoretical concentration dependence of the excess energy of mixing does result in improved agreement with the experimental results. However, the theoretical improvement introduces an extra theoretical parameter, and the analysis of the cloud point data becomes more complicated. The limited set of experimental data and the accuracy of the available experimental data make it impossible to evaluate the improved theory. Much larger and more accurate data sets would make it possible to evaluate the theory. However, other experimental data, such as scattering results in homogeneous solutions, would be more useful for determining the necessary theoretical parameter values independent from the experimental cloud point data that could then be predicted.

Nonrandom mixing effects become important with larger interaction energies (details in Appendix A.3.2). The Guggenheim theory was extended to ternary mixtures [71,72,73,74], but, unfortunately, determining the effect of nonrandom mixing on the excess thermodynamic functions requires iterative numerical procedures. Nevertheless, we can still come to some generic conclusions on the nonrandom mixing effects from the (LCT) x–expansion. From the x–expansion, we find that the zero-order term is purely entropic, the first-order term is of pure energic nature, and from the second order onwards in the x–expansion, both the energetic and entropic contributions contain $x^{i}$. Hence, in all generality, it can be concluded that from the second order onwards, the energetic parameters, $\Delta \epsilon_{ij}$ $\left(\chi_{IJ}\right)$, will influence both the excess entropy and energy of mixing, which is a direct consequence of nonrandom mixing. In the case of an end-functionalized homopolymer and a copolymer, the intramolecular properties, entailed in the intramolecular composition and the characteristic nearest-neighbor potential energies, will show up in the energy and entropy.

When nonrandom mixing effects become important, then the intramolecular details of the end-functionalized homopolymer and/or the copolymer composition (distribution) will also enter the excess entropy of mixing as well as the excess energy of mixing. However, these effects only become visible from the second-order improvement onwards (details in Appendix A.3.3). Possible practical criteria for estimating when nonrandom mixing effects become relevant are suggested in Appendix A.3.4.

The presented FH theory and the theoretical improvements provided by the Huggins and Guggenheim theories can only be put to detailed scrutiny when more and true thermodynamic cloud point data are available. We anticipate that the improved theories will also improve the correspondence between theory and experiments. Furthermore, other types of experimental data, e.g., concentration fluctuations that can be obtained from scattering experiments in a homogenous state measured as a function of concentration and temperature, will make it possible to establish if the theoretical improvements will indeed be as effective as hoped for and will make it possible to truly predict, e.g., the cloud point temperatures from experimental data that are different from the cloud point data itself.

### 4.4. Other Available Theories versus the Simple FH Theory for End-Functionalized Polymers

Numerous publications by Tanaka and collaborators deal with the importance of the hydration of the chain molecules and the association between chain molecules and their impact on the (LCST) phase behavior of these water-soluble homopolymers, copolymers, and end-functionalized polymers. In these theoretical works, the complex interplay between cooperative dehydration and association-induced phase separation is used to explain the observed phase behavior. Telechelic PEO and telechelic PNIPAM systems were studied in detail, showing the versatility of the theory [75]. In this work, the interaction parameters between PNIPAM and water units and telechelic chains and water were considered, but the interaction between PNIPAM and telechelic chains was not explicitly discussed. The theory was also applied to random copolymers [76]. In this work, the interaction parameters of the segments in a copolymer with water were taken to be identical, and the interaction parameter between the two distinct copolymer segments was set equal to zero.

In summary, the theory by Tanaka and collaborators is quite versatile, with an emphasis on the importance of cooperative dehydration and the associated effects on phase behavior. As far as we know, the direct application of the theory, including explicitly the effects of the different interaction parameters as discussed in this manuscript, has not (yet) been carried out.

Thermodynamic theories for associating systems, such as the Perturbed Chain–Statistical Associating Fluid Theory (PC-SAFT) [77,78,79], were developed based on Wertheim’s theoretical framework for the treatment of systems involving saturation interactions (the hydrogen bond is an important example of a saturation interaction) [80,81,82,83]. The PC-SAFT has been applied to many experimental systems, including aqueous polymer solutions of homopolymers and copolymers. In principle, the theory is also applicable to end-functionalized homopolymers, but we are not aware of any application of the theory to this system. The PC-SAFT is also a versatile theory and is also able to predict the phase behavior of, e.g., aqueous solutions of PNIPAM and PEO. The phase behavior of the two systems is predicted to be in good agreement with experimental data [84,85].

A salient point when comparing these two different lines of approach is that although the theory by Tanaka and coworkers and the PC-SAFT are significantly different in terms of their theoretical approach, both can predict the phase of aqueous solutions of polymers. To our knowledge, the effect of end groups has not been discussed in detail in the context of these theories, but an extension of the theories taking end groups into account is feasible. The theoretical considerations developed in this manuscript are useful for the incorporation of end groups in the more sophisticated theory by Tanaka, the PC-SAFT, or similar Wertheim-based theories.

## 5. Conclusions

The FH theory amended for end groups confirms the validity of the excess entropy of mixing. In all the investigated cases, the effect of end groups is only present in the FH interaction parameters or functions.

As the interaction energies are not too large, the intramolecular details of an end-functionalized homopolymer (i.e., nonrandom mixing effects) are trivial and thus do not yet enter the excess entropy of mixing and the excess energy of mixing. Therefore, the random mixing assumption used is still applicable with the $\chi_{0}$ and the renormalized $\chi_{1}$ parameters, partially taking care of the theoretical improvement.

The predicted results are in reasonable agreement with the measured results, allowing for a clear understanding of the end group effects on the cloud points of polymers in aqueous solutions. This is confirmed by the theory that more hydrophilic end groups increase $T_{cp}$, whereas less hydrophilic and hydrophobic end groups decrease $T_{cp}$. End groups only show pronounced effects at low polymer molar masses, and all the cloud point curves finally converge to $T_{cp}^{\infty }\left(\phi_{cp}\right)$ at sufficiently high polymer molar masses. This explains very well the inverse dependence, invariance, and direct dependence of $T_{cp}$ on the polymer molar mass experimentally observed so far.

However, it must be noted that the ultimate scrutiny of the simple FH theory and the suggested improved theories must await the measurement of truly thermodynamic cloud points; the available cloud points are merely estimations of the thermodynamic cloud point for which the deviation to the true cloud point cannot be established with sufficient accuracy.

This finding suggests a novel approach for a better rationalization of the end group effects since the effects of end groups and polymer molar mass on $T_{cp}$ are additive and can be separated for further study. The application of the theory is not limited to thermoresponsive polymers in aqueous solutions but applies equally well to all end-functionalized homopolymers and statistical copolymers in organic solvents. Moreover, the theory is also applicable to UCST phase behavior, which is obtained when $\chi_{1}>0$.

## Figures and Tables

**Table 1:** The limiting values of $\chi_{cp}\left(\phi_{cp},\;s_{P}\to \infty \right)$ and the polynomial coefficients $B_{i}$ in $\left(\chi_{cp}\left(\phi_{cp},s_{P}\right)-\chi_{cp}\left(\phi_{cp},s_{P}\to \infty \right)\right)=10^{\left(\overset{6}{\underset{i=0}{\sum }}B_{i}{\left(\log s_{P}\right)}^{i}\right)}$ for $\phi_{cp}=0.05,\;0.01,\;0.005,\;\mathrm{and}\;0.001$.

$\phi_{cp}$	$\chi_{cp}\left(s_{P}\to \infty \right)$	B0	B1	B2	B3	B4	B5	B6
0.05	0.517318	0.4415	−0.87889	0.16559	−0.08219	0.02766	−0.0061	5.24856 × 10^−4^
0.01	0.503359	0.62228	−0.89899	0.12377	−0.01639	−0.00327	0.00108	−9.87525 × 10^−5^
0.005	0.501673	0.6859	−0.91305	0.12385	−0.01107	−0.00516	0.00141	−1.15139 × 10^−4^
0.001	0.500334	0.80834	−0.93836	0.11805	0.00151	−0.00946	0.00209	−1.52861 × 10^−4^

**Table 2:** Coefficients obtained from FH linear fits (depicted in Figure 5) using Equation (9) with $a_{1}=\chi_{OE_{1}}M_{E_{1}}$ for PNIPAM polymers containing various R groups.

R Groups	Coefficients
	$a_{1}$ (kDa)
-NH_2_	−0.008 ± 0.009
-OEt	0.12 ± 0.01
-NHPh	0.22 ± 0.01
-IBN	0.43 ± 0.04
-CONH_2_	0.69 ± 0.05
-Py	0.71 ± 0.02
-CONH-Tr	0.92 ± 0.05

**Table 3:** Coefficients obtained from FH linear fits (depicted in Figure 9) using Equation (9) with $a_{1}=\chi_{OE_{1}}M_{E_{1}}$ for PTEGSt polymers containing various end groups.

End Groups	Coefficients
	$a_{1}$(kDa)
-Ph	0.47 ± 0.04 (fitted)
-ON	0.53 ± 0.02 (fitted)
-Ph and -ON	1.00 ± 0.05 (predicted)

**Table 4:** Coefficients obtained from FH linear fits (depicted in Figure 13) using Equations (9) and (11) for PHTrEGSt polymers containing various end groups.

End Groups	Coefficients
	$a_{1}\;$(kDa)
-ON	0.41 ± 0.03 (fitted)
-Ph and -ON	0.74 ± 0.01 (fitted)
-Ph	0.33 ± 0.03 (predicted)

**Figure A1:**
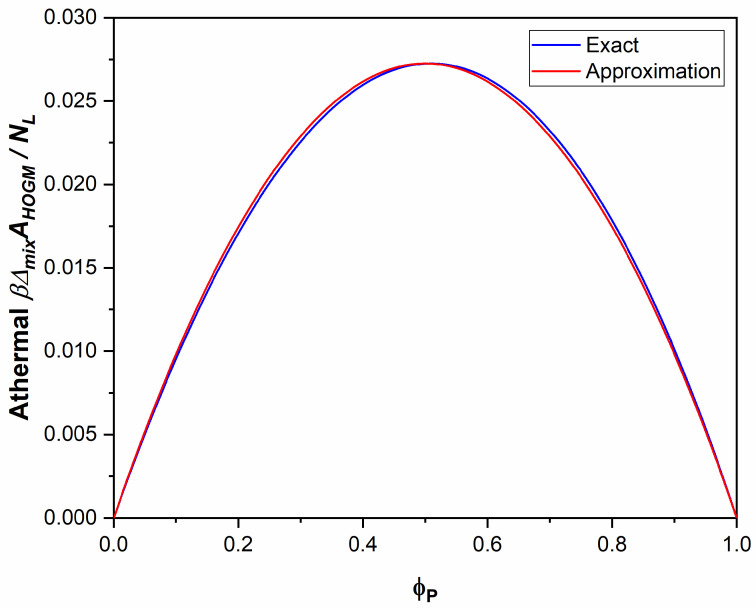
Exact value of $\beta \Delta_{mix}A_{HOGM}^{Athermal}/N_{L}$ and the approximate quadratic concentration dependence of $\alpha_{m}\;\phi_{P}\left(1-\phi_{P}\right)$ on $\phi_{P}$ for z=10 and $s_{P}=100$.

**Figure A2:**
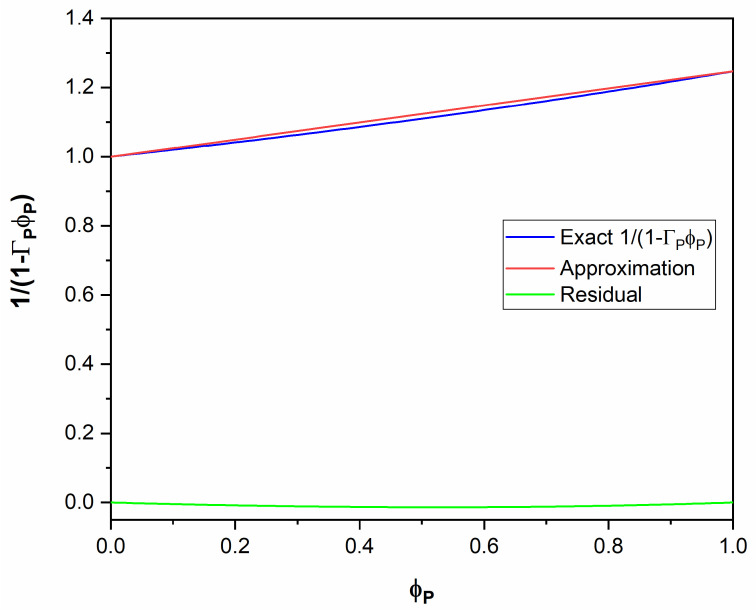
Exact value of the concentration dependence, $1/\left(1-\Gamma_{P}\phi_{P}\right)$, the linear approximation, and the residual versus $\phi_{P}$ for z=10 and $s_{P}=100$.

## Data Availability

Data are contained within the article.

## Supplementary Materials

The following supporting information can be downloaded at https://www.mdpi.com/article/10.3390/polym16040563/s1: Table S1: Properties of PNIPAM polymers with various R groups and different molar mass in aqueous solution; Table S2: $T_{cp}$ versus $M_{n}$ of PTEGSt containing various chain ends; Table S3: $T_{cp}$ versus $M_{n}$ of PHTrEGSt containing various chain ends; Table S4: R groups (in original papers) versus end groups (in this work) in PNIPAM polymers; Figure S1: χ vs. $1/M_{n}$ for the hypothetical invariant end-functionalized PNIPAM system; figures and tables showing the 0.95 confidence interval and the statistical parameters’ t-statistics and P-values for the FH parameter values for homopolymers and end-functionalized homopolymers.

## Appendix A

Although liquids live in a continuum and do not reside on a lattice, lattice models still hold relevance for the discussion of the properties of polymer solutions and mixtures. In this work, the Flory–Huggins (FH) excess Gibbs energy of mixing given by Equation (3) in combination with Equation (6) was used to interpret the cloud point temperature data of *α*, *ω*-end-functionalized homopolymers in aqueous solutions.

In the following, we discuss the nature of the approximation of the FH theory, existing theoretical improvements to the FH theory, the relationships between them, and the consequences of the different improvements. This will be carried out for a linear homopolymer without end groups using the standard FH theory for statistical copolymers and end-functionalized homopolymers.

### Appendix A.1. FH Theory for Solutions of Linear Homopolymers without End Groups, Statistical Terpolymers, and α, ω–End-Functionalized Homopolymers

Consider a mixture of $N_{P}$ linear polymer molecules, each with $s_{P}$ lattice segments and $N_{O}$ solvent molecules, each taking one lattice site, placed on a cubic lattice with coordination number z. The polymer segments and solvent molecules interact via constant nearest-neighbor potential energies, $-\epsilon_{PO},-\epsilon_{PP},\;\mathrm{and}-\epsilon_{OO}$. The FH excess Helmholtz energy of the mixing of the system, $\Delta_{mix}A_{FH}$, is given by the following:

(A1) $$\beta \Delta_{mix}A_{FH}/N_{L}=\beta \Delta_{mix}G_{FH}/N_{L}=\phi_{O}\ln\phi_{O}+\frac{\phi_{P}}{s_{P}}\ln\phi_{P}+\chi \phi_{O}\phi_{P}$$

where $\beta =1/k_{B}T$, $N_{L}=N_{P}s_{P}+N_{O}$ is the total number of lattice sites, $\phi_{P}\;\left(\phi_{O}\right)$ is the polymer (solvent) volume fraction, and χ=zβΔϵ/2, $\Delta \epsilon =\left(2\epsilon_{PO}-\epsilon_{PP}-\epsilon_{OO}\right)$. The equality of $\Delta_{mix}A_{FH}$ and $\Delta_{mix}G_{FH}$ follows from $\Delta_{mix}V_{FH}=0$.

In the FH theory, an *α*, *ω*-end-functionalized homopolymer is considered a particular case of a copolymer. The same approximations that give rise to $\Delta_{mix}A_{FH\;}$ for the linear homopolymer without end groups lead for a statistical (*A*,*B*,*C*)-terpolymer with distinct repeat units to the conclusion that the excess Helmholtz energy of mixing is also given by Equation (A1) with the stipulation that the effect of the different repeat units is not seen in the excess entropy of mixing but only in the energetic contribution to the excess Helmholtz energy of mixing in an *effective* FH interaction function χ, explicitly depending *only* on the *copolymer composition* [44,45,46,47,48], viz.

$\chi =\left(y_{A}\chi_{0A}+y_{B}\chi_{OB}+y_{C}\chi_{OC}-y_{A}y_{B}\chi_{AB\;}-y_{A}y_{C}\chi_{AC\;}-y_{B}y_{C}\chi_{BC\;}\right)$, where $y_{I}$ is the volume fraction of comonomer $I=\left(A,B,C\right)$ in the terpolymer and $y_{A}+y_{B}+y_{C}=1$.

When applying the terpolymer theory to an *α*, *ω*-end-functionalized homopolymer with distinct end groups at each end, the terpolymer composition variables must be mapped to the different units in the end-functionalized polymer. The total number of segments in a polymer chain is $s_{P}$. The number of middle segments is mapped to a copolymer component of type *A*; $s_{M}\equiv s_{A}=s_{P}-s_{B}-s_{C};$ and $y_{M}\equiv y_{A}=\left(s_{P}-s_{B}-s_{C}\right)/s_{P}$. The end group $E_{1}$ is mapped to a copolymer unit of type *B*, with $y_{E_{1}}\equiv y_{B}=s_{B}/s_{P}$, and the end group $E_{2}$ is mapped to a copolymer unit of type *C*, with $y_{E_{2}}\equiv y_{C}=s_{C}/s_{P}$. The volume fractions $y_{E_{1}},\;y_{E_{2}},\;\mathrm{and}\;y_{M}$ can also be related directly to the molar mass of the polymer. The number of lattice sites taken by the polymer in a solvent is $s_{P}=Mv_{P}/V_{L}$, where M is the molar mass of the polymer, including all repeat units, i.e., the middle units as well as the end units (possibly either of type $E_{1}$ or type $E_{2}$); $v_{P}=1/\rho_{P}$ is the specific volume of the polymer; $\rho_{P}$ is its density; and $V_{L}$ is the molar volume of the lattice site, which is taken as being equal to the molar volume of the solvent. The number of lattice sites taken by the end unit of type $E_{i}$ can be calculated from $s_{E_{i}}=n_{E_{i}}M_{E_{i}}v_{P}/V_{L},$ where $M_{E_{i}}$ is the molar mass of the complete end group $E_{i}$. The constant $n_{E_{i}}$ is introduced so that the general result can be reduced to a standard FH homopolymer $\left(n_{E_{1}}=n_{E_{2}}=0\right)$ and a mono-end-functionalized homopolymer $\left(n_{E_{1}}=1,\;n_{E_{2}}=0\right)$. The fraction of lattice sites taken by the end unit(s) $E_{i}$ per chain is $y_{E_{i}}=\frac{s_{E_{i}}}{s_{P}}=\left(n_{E_{i}}M_{E_{i}}v_{P}/V_{L}\right)/\left(Mv_{P}/V_{L}\right)=\frac{n_{E_{i}}M_{E_{i}}}{M}$.

Note also that we only need the density of the polymer chain itself (which could vary with the molar mass).

Thus, the excess Helmholtz energy of mixing is once again given by Equation (A1), and the effect of the end functionalization of the polymer is not seen in the excess entropy of mixing but once again only in the *effective* FH interaction function χ, depending on the molar masses of the end groups and the molar mass of the entire end-functionalized homopolymer as well as the involved $\chi_{IJ}$ FH parameters $\left(\chi_{OM},\;\chi_{OE_{1}},\;\chi_{OE_{2}},\;\chi_{ME_{1}},\;\chi_{ME_{2}},\;\chi_{E_{1}E_{2}}\right)$ as shown in Equation (6) in the main text.

### Appendix A.2. Lattice Theories beyond FH Theory for Linear Homopolymers without End Groups

#### Appendix A.2.1. Huggins, Guggenheim, and Lattice Cluster Theories

The FH theory is probably the best known approximation of the exact results of a lattice model, although more accurate theoretical approximations are available, such as the expressions for the excess Gibbs energy of mixing given by Huggins [32,33], Orr [52], Guggenheim [53,54,55], and Miller [56,57].

Almost half a century later, Freed and coworkers introduced the Lattice Cluster Theory (LCT), constituting an exact theoretical framework for lattice models, facilitating the systematic construction of improved approximations [51,58,59,60,61,62,63,64,65,66,67,68].

Moreover, the LCT can also deal with more complex lattice models for polymer systems. For example, the LCT has been used to study and explain the effects of monomer structure and compressibility on the thermodynamic properties of homopolymers, random and diblock copolymers, and telechelic polymer systems [58,63,86,87,88,89,90,91,92,93,94,95].

#### Appendix A.2.2. Lattice Cluster Theory for Linear Homopolymers (No Distinct End Groups) in Solvents

For the simplest linear polymer considered in Section A.1, the excess Helmholtz energy of the mixing of the system, $\Delta_{mix}A$, is written in the LCT formula as a double expansion of the (dimensionless) interaction energy, x≡βΔϵ, and the inverse of the lattice coordination number, $z^{-1}$ [51], viz.

(A2) $$\beta \Delta_{mix}A/N_{L}=\underset{i=0}{\sum }a_{i}x^{i}=\beta \Delta_{mix}A_{0}/N_{L}+\underset{i=1}{\sum }a_{i}x^{i}$$

In the athermal limit ($\frac{1}{k_{B}T}=0,\;i.e.,\;T\to \infty \;K$), the zero-order coefficient, $a_{0}$, is the only term surviving in the expansion and is thus equal to the athermal excess Helmholtz energy of mixing, $\Delta A_{0},$ which is of pure entropic origin; thus, $a_{0}\equiv \;\beta \Delta_{mix}A_{0}/N_{L}$. The composition-dependent expansion coefficients $a_{i}\left(\phi_{P}\right)$ are, on their own, obtained as an expansion in $z^{-1}$.

(A3) $$a_{i}=\underset{j=0}{\sum }\zeta_{j}^{\left(i\right)}\left(\phi_{P}\right){\left(z^{-1}\right)}^{j}\;and\;a_{0}=\underset{j=0}{\sum }\zeta_{j}^{\left(0\right)}\left(\phi_{P}\right){\left(z^{-1}\right)}^{j}$$

where $\zeta_{j}^{\left(i\right)}\left(\phi_{P}\right)$ are polynomial expressions in $\phi_{P}$.

The LCT framework has an enormous ability for systematic improvement and the ease of constructing more complex lattice models for polymer systems. This conceptual and principal advantage, however, also comes at the cost of the increasing complexity involved in the calculations of higher-order coefficients in the cluster expansion [58].

#### Appendix A.2.3. Relation between the LCT and the FH Theory

Madden et al. showed that the zero-order term, $\zeta_{0}^{\left(0\right)}\left(\phi_{P}\right)$, in the athermal excess Helmholtz energy of mixing is identical to the (athermal) FH excess entropy of mixing [51], i.e., $\beta \Delta_{mix}A_{0\;}/N_{L}=\beta \Delta_{mix}A_{FH}^{Athermal}/N_{L}=\phi_{O\;}\ln\phi_{O}+\frac{\phi_{P}}{s_{P}}\ln\phi_{P}$.

Moreover, limiting the LCT expansion (Equations (A2) and (A3)) to only the first-order term, $a_{1}=\underset{j=0}{\sum }\zeta_{j}^{\left(1\right)}\left(\phi_{P}\right)$, and retaining only the zero-order term in the $z^{-1}$ expansion, i.e., $a_{1}=\zeta_{0}^{\left(1\right)}\left(\phi_{P}\right)=\frac{z}{2}\phi_{O}\phi_{P}$, gives for $\frac{\beta \Delta_{mix}A_{1\;}}{N_{L}}=\frac{z}{2}\phi_{O}\phi_{P}x=\frac{z}{2}\phi_{O}\phi_{P}\beta \Delta \epsilon =\chi \phi_{O}\phi_{P}$ the FH expression for the excess energy (enthalpy) of mixing in Equation (A1) [51].

Madden et al. thus demonstrated that in the framework of the LCT, the FH theory is a first-order perturbation theory in x with respect to an athermal reference system, taking only the zero-order terms in the $z^{-1}$ expansion for both $\frac{\beta \Delta_{mix}A_{0\;}}{N_{L}}$ and $\frac{\beta \Delta_{mix}A_{1\;}}{N_{L}}$.

#### Appendix A.2.4. Relation between the LCT and the Huggins Theory

Huggins obtained in his derivation of what we now call the FH theory a more accurate approximation for the excess entropy of mixing [32,33]. Huggins took into account the connectivity of the segments (the middle segments always have at least two nearest-neighbor lattice positions occupied by the two neighboring segments in the chain, whereas for the end segments, only one nearest-neighbor lattice site is taken by the nearest-neighboring segment of the end segment), and the number of available configurations on the lattice is smaller than assumed in the FH theory, leading to a reduction in the excess entropy of mixing. The athermal excess Helmholtz energy of mixing, according to Huggins, is as follows:

(A4) $$\beta \Delta_{mix}A_{Huggins}^{Athermal}/N_{L}=\beta \Delta_{mix}A_{FH}^{Athermal}/N_{L}+\beta \Delta_{mix}A_{HOGM}^{Athermal}/N_{L}$$

(A5) $$\beta \Delta_{mix}A_{HOGM}^{Athermal}/N_{L}=\frac{1}{\gamma }\left(\left(1-\Gamma_{P}\right)\phi_{P}\ln\left(1-\Gamma_{P}\right)-\left(1-\Gamma_{P}\phi_{P}\right)\ln\left(1-\Gamma_{P}\phi_{P}\right)\right)$$

where $\gamma =2z^{-1},\;\Gamma_{P}=\gamma \left(1-1/s_{P}\right),$ and the subscript HOGM refers to Huggins, Orr, Guggenheim, and Miller, reminding us that the Huggins approximation was also obtained independently by Orr [52], Guggenheim [53,54,55], and Miller [56,57].

Accounting in the same manner for the chain connectivity, the excess energy of mixing becomes the following:

(A6) $$\frac{\beta \Delta_{mix}E_{Huggins}}{N_{L}}=\chi \phi_{O}\phi_{P}\frac{\left(1-\Gamma_{P}\right)}{\left(1-\Gamma_{P}\phi_{P}\right)}=\frac{\beta \Delta_{mix}E_{FH}}{N_{L}}\frac{\left(1-\Gamma_{P}\right)}{\left(1-\Gamma_{P}\phi_{P}\right)}$$

Combining Equations (A4)–(A6), the Huggins approximation for the excess Helmholtz energy of mixing is as follows:

(A7) $$\begin{array}{l} \beta \Delta_{mix}A_{Huggins}/N_{L}=\beta \Delta_{mix}A_{Huggins}^{Athermal}/N_{L}+\beta \Delta_{mix}E_{Huggins}/N_{L}\\ \;\;\;\;\;\;\;=\beta \Delta_{mix}A_{FH}^{Athermal}\\ \;\;\;\;\;\;\;+\frac{1}{\gamma }\left(\left(1-\Gamma_{P}\right)\phi_{P}\ln\left(1-\Gamma_{P}\right)-\left(1-\Gamma_{P}\phi_{P}\right)\ln\left(1-\Gamma_{P}\phi_{P}\right)\right)\\ \;\;\;\;\;\;\;+\frac{\beta \Delta_{mix}E_{FH}}{N_{L}}\frac{\left(1-\Gamma_{P}\right)}{\left(1-\Gamma_{P}\phi_{P}\right)} \end{array}$$

Note that in the limit z→ ∞, we obtain γ→0 and $\Gamma_{P}\to 0,$ and the excess Helmholtz energy of mixing, $\frac{\beta \Delta_{mix}A_{FH}}{N_{L}},$ is recovered.

The Huggins approximations for the excess entropy of mixing and excess energy of mixing give a substantial improvement in comparison with the available simulation results [51,58,69,70].

#### Appendix A.2.5. Relation between the LCT and the Guggenheim Theory

The theory developed by Guggenheim considers the effects of nonrandom mixing due to differences in the nearest-neighbor potential energies. The effects of nonrandom mixing are expressed in a so-called quasi-chemical equation, and in the present case of a single energy scale, $\beta \Delta \epsilon =\beta \left(2\epsilon_{PO}-\epsilon_{PP}-\epsilon_{OO}\right)\;\left(\mathrm{or}\;\chi =\beta \Delta \epsilon /2\right)$, the excess Helmholtz energy of mixing can still be presented in closed form [53,54,55], i.e.,

(A8) $$\begin{array}{l} \beta \Delta_{mix}A_{Guggenheim}/N_{L}\\ \;\;\;\;\;\;=\beta \Delta_{mix}A_{Huggins}^{Athermal}/N_{L}\\ \;\;\;\;\;\;+\frac{1}{\gamma }\left(\phi_{O}\ln\left(\left(1-\kappa \theta_{P}\right)/\theta_{O}\right)+\left(1-\Gamma_{P}\right)\phi_{P}\ln\left(\left(1-\kappa \theta_{O}\right)/\theta_{P}\right)\right) \end{array}$$

with $\kappa =2/\left(\alpha +1\;\right),\alpha ={\left(1+4\theta_{O}\theta_{P}\left(K^{2}-1\right)\right)}^{\frac{1}{2}}$, $K=\exp\left(\beta \Delta \epsilon /2\right)$, $\theta_{P}=\phi_{P}\left(1-\Gamma_{P}\right)/\left(1-\Gamma_{P}\phi_{P}\right),$ and $\theta_{O}=1-\theta_{P}$.

As reported by Freed and coworkers, the quasi-chemical theory by Guggenheim gives very good agreement with the simulation data [51,58].

#### Appendix A.2.6. Relation between the Huggins Theory and the Guggenheim Theory

The athermal result $\beta \Delta_{mix}A_{Guggenheim}^{Athermal}/N_{L}$ is obtained for K=1, for which κ=1 and the two last terms on the right-hand side in Equation (A8) are identical zero. Hence, in the athermal case, the Guggenheim theory is identical to $\beta \Delta_{mix}A_{Huggins}^{Athermal}/N_{L}$.

Furthermore, for βΔϵ→0, we can linearize K=1+βΔϵ, with, in the same approximation, $\alpha =1+2\beta \Delta \epsilon \theta_{O}\theta_{P}$ and $\kappa =1-\beta \Delta \epsilon \theta_{O}\theta_{P},$ and finally, the Guggenheim expression for the excess Helmholtz energy of mixing is as follows:

$$\begin{array}{l} \beta \Delta_{mix}A_{Guggenheim}/N_{L}=\beta \Delta_{mix}A_{Huggins}^{Athermal}/N_{L}+\frac{1}{\gamma }\beta \Delta \epsilon \left(\phi_{P}\left(1-\Gamma_{P}\right)\theta_{O}^{2}+\phi_{O}\theta_{P}^{2}\right)\\ \;\;\;\;\;\;=\beta \Delta_{mix}A_{Huggins}^{Athermal}/N_{L}+\frac{1}{\gamma }\beta \Delta \epsilon \frac{\phi_{O}\phi_{P}\left(1-\Gamma_{P}\right)}{\left(1-\Gamma_{P}\phi_{P}\right)}\\ \;\;\;\;\;\;=\beta \Delta_{mix}A_{Huggins}^{Athermal}/N_{L}+\frac{z}{2}\beta \Delta \epsilon \frac{\phi_{O}\phi_{P}\left(1-\Gamma_{P}\right)}{\left(1-\Gamma_{P}\phi_{P}\right)}\\ \;\;\;\;\;\;=\beta \Delta_{mix}A_{Huggins}^{Athermal}/N_{L}+\chi \frac{\phi_{O}\phi_{P}\left(1-\Gamma_{P}\right)}{\left(1-\Gamma_{P}\phi_{P}\right)}\\ \;\;\;\;\;\;=\beta \Delta_{mix}A_{Huggins}^{Athermal}/N_{L}+\beta \Delta_{mix}E_{Huggins}/N_{L} \end{array}$$

which is exactly the Huggins result. Thus, the Huggins results is a limiting case of the Guggenheim result that is obtained for sufficiently small βΔϵ, βΔϵ→0, where the random mixing assumption used by Huggins still applies.

#### Appendix A.2.7. Estimate of the Effects of $\Delta_{mix}A_{HOGM}^{Athermal}$ and $\Delta_{mix}E_{Huggins}$ on the FH Predictions

The Huggins approximation for the combinatorial entropy of mixing explicitly addresses the difference between the middle and end segments in a chain. For the systems studied in the simulations, $\Delta_{mix}A_{HOGM}^{Athermal}$ takes care of the largest part of the deviations between the FH entropy and the exact entropy obtained in the simulations. $\beta \Delta_{mix}A_{HOGM}^{Athermal}/N_{L}$ is presented in Figure A1 for $z=10\;\mathrm{and}\;s_{p}=100$.

The entropic term $\beta \Delta_{mix}A_{HOGM}^{Athermal}/N_{L}$ is, for all reasonable values of $z\;\left(z\geq 4\right)$, effectively approximated by the approximate quadratic concentration dependence $\frac{\beta \Delta_{mix}A_{HOGM}^{Athermal}}{N_{L}}≅\alpha_{m}\;\phi_{P}\left(1-\phi_{P}\right),$ where $\alpha_{m}$ is the maximum value in Figure A1, which occurs at $\phi_{P,m}$, which is very close but not exactly equal to $\phi_{P}=1/2$. The values of $\alpha_{m}$ and $\phi_{P,m}$ are as follows:

$$\alpha_{m}={\left(1-\Gamma_{P}\right)}^{-1/\Gamma_{P}}\left(\Gamma_{P}-1\right)\left(\Gamma_{P}\ln\left(\frac{{\left(1-\Gamma_{P}\right)}^{1-\frac{1}{\Gamma_{P}}}}{e}\right)-\left(e{\left(1-\Gamma_{P}\right)}^{1/\Gamma_{P}}+\Gamma_{P}-1\right)\ln\left(1-\Gamma_{P}\right)\right)/e\gamma \Gamma_{P}$$

and $\phi_{P,m}=\left(1-e^{\frac{\Gamma_{P}\ln\left(1-\Gamma_{P}\right)-\Gamma_{P}-\ln\left(1-\Gamma_{P}\right)}{\Gamma_{P}}}\right)/\Gamma_{P}$.

In the application of the FH theory to the experimental results, we use $\chi =\chi_{0}+\frac{\chi_{1}}{T},$ and the (approximate) Huggins entropic correction parameter, $\alpha_{m}$, is absorbed in $\chi_{0}$ when fitting the experimental data.

For the excess energy of mixing, $\beta \Delta_{mix}E_{Huggins}/N_{L}=\frac{\chi_{1}}{T}\phi_{O}\phi_{P}\left(\frac{\left(1-\Gamma_{P}\right)}{\left(1-\Gamma_{P}\phi_{P}\right)}\right),$ the effect of the connectivity rescales the bare value of the FH $\chi_{1}$ to $\left(1-\Gamma_{P}\right)\chi_{1}$, and this effect is automatically taken care of in the comparison with the experimental results when fitting the $\chi_{1}$ parameter to the experimental data. However, the additional concentration dependence, $1/\left(1-\Gamma_{P}\phi_{P}\right),$ is not accounted for in the simple FH theory. To estimate the importance of this concentration dependence, we rewrite it as follows:

(A9) $$1/\left(1-\Gamma_{P}\phi_{P}\right)=1+\phi_{P}\Gamma_{P}/\left(1-\Gamma_{P}\right)-\phi_{P}\left(1-\phi_{P}\right)\Gamma_{P}^{2}/\left(\left(1-\Gamma_{P}\right)\left(1-\Gamma_{P}\phi_{P}\right)\right)$$

where the first two terms on the right-hand side present the linear interpolation between the values of $1/\left(1-\Gamma_{P}\phi_{P}\right)$ at $\phi_{P}=0\;\mathrm{and}\;1,$ and the third term is the exact residual part. Figure A2 depicts the concentration dependence, $1/\left(1-\Gamma_{P}\phi_{P}\right),$ and the decomposition in the linear and residual terms for z=10 and $s_{P}=100.$ For all reasonable values of z, the linear approximation to $1/\left(1-\Gamma_{P}\phi_{P}\right)$ gives a good first-order approximation, and the residual term is significantly smaller.

#### Appendix A.2.8. Summary of Improvements and Relation to the FH Theory

For a linear homopolymer in a solution, the linearized Guggenheim theory and the Huggins theory are identical and are successful improvements to the FH theory. The Guggenheim and Huggins theories have the advantage that they give closed expressions that are valid over the complete composition and temperature ranges, whereas (perturbation) expansions have the potential to give spurious results when the truncated expansion is used outside its domain of applicability.

A closer inspection of the theoretical corrections to the excess Helmholtz energy of mixing shows that the corrections are small but relevant. The athermal entropy correction is effectively taken care of by the empirical entropy FH parameter $\chi_{0}$. The Huggins correction to the excess energy of mixing modifies the FH parameter $\chi_{1}$ to $\chi_{1}\left(1-\Gamma_{P}\right)$ and, in addition, the $1/\left(1-\Gamma_{P}\phi_{P}\right)$ concentration dependence is introduced. This is well known, and the Huggins theory improves the predicted critical conditions and the width of the binodal and spinodal curves significantly compared to the FH theory [96]. These are important improvements, but in the next paragraph, it will become clear that these quantitative improvements leave the conclusions intact that are reached concerning the importance of entropy and energy for end-functionalized homopolymers.

### Appendix A.3. Statistical Terpolymers and End-Functionalized Homopolymers in a Solvent

#### Appendix A.3.1. Huggins and Linearized Guggenheim Theories

In the Huggins theory, the intramolecular composition of a statistical copolymer is only exposed in the interaction energy and not in the entropy. Hence, the excess Helmholtz energy of mixing is also given by the following: (Equation (A7))

$$\begin{array}{l} \beta \Delta_{mix}A_{Huggins}/N_{L}\\ \;\;\;\;\;\;=\phi_{O}\ln\phi_{O}+\frac{\phi_{P}}{s_{P}}\ln\phi_{P}\\ \;\;\;\;\;\;+\frac{1}{\gamma }\left(\left(1-\Gamma_{P}\right)\phi_{P}\ln\left(1-\Gamma_{P}\right)-\left(1-\Gamma_{P}\phi_{P}\right)\ln\left(1-\Gamma_{P}\phi_{P}\right)\right)\\ \;\;\;\;\;\;+\chi \phi_{O}\phi_{P}\left(\frac{\left(1-\Gamma_{P}\right)}{\left(1-\Gamma_{P}\phi_{P}\right)}\right) \end{array}$$

with χ identical to the *effective* χ found in the FH theory, i.e., $\chi =\left(y_{A}\chi_{0A}+y_{B}\chi_{OB}+y_{C}\chi_{OC}-y_{A}y_{B}\chi_{AB\;}-y_{A}y_{C}\chi_{AC\;}-y_{B}y_{C}\chi_{BC\;}\right)$.

Also, in the application of the copolymer theory to an *α*, *ω*-end-functionalized homopolymer in a solution, the mapping of the copolymer composition variables to the molar masses of the polymer and the end groups remains the same as in the FH theory (Equation (6) in the main text).

It must be noted that the Huggins theory and the linearized Guggenheim theory take the effect of the end segments explicitly into account, and they manage this with surprising success. This success is most probably related to a fortuitous cancellation of errors [97].

#### Appendix A.3.2. Nonrandom Mixing Guggenheim Theory

In the case of the solutions of a statistical copolymer or an end-functionalized homopolymer with chemically distinct middle repeat units and the same end group at both ends, three χ’s and the same number of quasi-chemical equations are required in the Guggenheim theory. For an *α*, *ω*-end-functionalized homopolymer (and a statistical terpolymer), the situation requires six χ parameters and quasi-chemical equations.

The Guggenheim theory was extended to ternary mixtures [71,72,73,74], but, unfortunately, the effect of nonrandom mixing on the excess thermodynamic functions requires iterative numerical procedures. Nevertheless, we can still come to some generic conclusions on the nonrandom mixing effects from the (LCT) x–expansion (Equation (A2)).

#### Appendix A.3.3. General Statement on the Effect of Nonrandom Mixing

The LCT x–expansion for the Helmholtz energy (Equation (A2)) is an expansion with respect to the inverse temperature of the athermal reference state $\beta \Delta_{mix}A_{0}/N_{L}($the zero-order coefficient, $a_{0}$).

The x–expansion for the excess Helmholtz energy of mixing is in fact very general and not specific to the LCT (the $z^{-1}–$expansion of the $a_{i}$ coefficients is unique to the LCT though). Focusing on Equation (A2) and using the observation that all $a_{i}$ are independent of temperature combined with general thermodynamic principles, we can determine from Equation (A2) an expression for the excess energy of mixing:

(A10) $$\frac{\beta \Delta E_{mix}}{N_{L}}=\beta {\left.\frac{\partial }{\partial \beta }\left(\frac{\beta \Delta_{mix}A}{N_{L}}\right)\right|}_{V}=\beta {\left.\frac{\partial }{\partial \beta }\left(\underset{i=1}{\sum }a_{i}x^{i}\;\right)\right|}_{V}=\beta \underset{i=1}{\sum }ia_{i}x^{i-1}\Delta \epsilon =\underset{i=1}{\sum }e_{i}x^{i}$$

with $e_{i}\equiv ia_{i}$.

And for the excess entropy of mixing,

(A11) $$\begin{array}{l} \frac{\Delta S_{mix}}{k_{B}N_{L}}=\frac{\beta \left(\Delta_{mix}E-\Delta_{mix}A\right)}{N_{L}}=\underset{i=1}{\sum }ia_{i}x^{i}-\frac{\beta \Delta_{mix}A_{0}}{N_{L}}-\underset{i=1}{\sum }a_{i}x^{i}\\ \;\;\;\;\;\;=-\frac{\beta \Delta_{mix}A_{0}}{N_{L}}+\underset{i=1}{\sum }a_{i}\left(i-1\right)x^{i}=-\frac{\beta \Delta_{mix}A_{0}}{N_{L}}+\underset{i=1}{\sum }s_{i}x^{i} \end{array}$$

with $s_{i}\equiv \left(i-1\right)a_{i}$.

From the x–expansion, we find that the zero-order term is purely entropic, the first-order term is of a purely energic nature, and from the second-order term onwards in the x–expansion, both the energy and entropic contributions contain $x^{i}$. For instance, for the second-order term, we have $\frac{\beta \Delta_{mix}A_{2}}{N_{L}}=a_{2}x^{2}=a_{2}{\left(\beta \Delta \epsilon \right)}^{2}$, $\frac{\beta \Delta_{mix}E_{2}}{N_{L}}=2a_{2}x^{2}=2a_{2}{\left(\beta \Delta \epsilon \right)}^{2},$ and $\frac{\beta \Delta_{mix}S_{2}}{N_{L}}=a_{2}x^{2}=a_{2}{\left(\beta \Delta \epsilon \right)}^{2}$.

Hence, in all generality, it can be concluded that from the second order onwards, the energetic parameters, $\Delta \epsilon_{ij}$ $\left(\chi_{IJ}\right)$, will influence both the excess entropy and energy of mixing, a direct consequence of nonrandom mixing. In the case of an end-functionalized homopolymer and a copolymer, the intramolecular properties, entailed in the intramolecular composition and the characteristic nearest-neighbor potential energies, will show up in energy and entropy.

#### Appendix A.3.4. Summary and Consequences of Improvements for the Functionalized Homopolymers

When the interaction energies $\left(\beta \Delta \epsilon \right)$ are sufficiently small, the Huggins theory and the linearized Guggenheim theory for a statistical terpolymer or an end-functionalized homopolymer in a solvent remain the same as these theories for the case of a linear homopolymer without end groups in a solvent, but the FH interaction function χ now becomes the *effective* χ, as in the FH theory, which takes into account the intramolecular composition variables. The Huggins and the linearized Guggenheim theories still successfully improve the FH theory with small but relevant corrections to the excess Helmholtz energy of mixing.

When the interaction energies $\left(\beta \Delta \epsilon \right)$ are sufficiently large (i.e., the effect of nonrandom mixing becomes significant), they will present and influence both the excess entropy and energy of mixing from the second order onwards in the LCT x–expansion for the Helmholtz energy. As a result, the intramolecular properties of a copolymer or an end-functionalized homopolymer in a solution will show up in both the entropy and energy.

A general approach for estimating the threshold beyond which the nonrandom mixing theory will become less effective could be based on the series expansion in Equation (A2) defined in Appendix A.2.2. From that series expansion, we deduced in all generality that the effects of nonrandom mixing will become discernible from the second-order term in x. Nonrandom mixing effects will become important when the term $a_{2}x^{2}$ is of the same order as the sum of the previous terms, i.e., $a_{0}+a_{1}x$. For this, of course, we must make an estimate of the factor $a_{2},$ and we can use, e.g., the Guggenheim theory or any other theory available that includes nonrandom mixing effects.

A useful analysis of the Guggenheim theory was carried out for small molecules by Guggenheim (see paragraph 4.24 of chapter 4 in [55]). The conclusion was that the Guggenheim excess Helmholtz energy expression is a useful approximation of the exact excess Helmholtz energy expression for values of x≤ ½, which roughly correspond to the temperature domain of a one-phase homogeneous mixture state up to the liquid–liquid (L-L) critical temperature of the mixture. A similar conclusion was reached for polymer solutions based on a numerical evaluation of the number of contacts assuming random mixing and the number of contacts calculated by the full Guggenheim theory (see paragraph 11.14 of chapter 11 in [55]). Again, in the one-phase homogeneous mixture state up to the L-L critical temperature, the random mixing theory will be a good approximation. Moreover, when the L-L miscibility gap is rather flat, as often observed in the LCST behavior of aqueous polymer solutions, the random mixing approximation will be good up to the cloud point temperature at any composition.
