# Supplementary material for: Effect of End Groups on the Cloud Point Temperature of Aqueous Solutions of Thermoresponsive Polymers: An Inside View by Flory–Huggins Theory
Source: Polymers (Basel). 2024 Feb 19;16(4):563. doi: 10.3390/polym16040563 (PMC10893037; doi:10.3390/polym16040563)

Supplementary Materials

S.1. Experimental Data from the Original References

Table S1. Properties of PNIPAM polymers with various R groups and different molar mass in aqueous solution [13,17,18,29].

R	$M_{n,th}$ (kDa)[a]	$M_{n,NMR}$ (kDa)[b]	$M_{n,MS}$ (kDa)[c]	M_w (kDa)[d]	PDI[e]	T_{cp} (K)[f]
$-NH_2$	3.0				1.07	318.45
	4.5				1.07	313.85
	8.8				1.08	309.45
	16.3				1.09	307.55
-NH-*i*-Pr	3.1				1.15	316.05
	4.9				1.11	313.45
	8.8				1.14	309.15
	19.2				1.12	307.25
-OMe		2.8			1.07	316.15
		5.0			1.15	312.05
		6.5			1.09	309.45
		6.7			1.13	309.55
		10.9			1.11	308.65
		15.7			1.13	307.75
		26.5			1.16	306.45
OEt		3.0			1.19	313.75
		4.3			1.06	310.95
		7.3			1.10	308.35
		15.2			1.13	306.45
-NHPh		3.2			1.10	310.55
		4.8			1.08	308.25
		9.3			1.11	307.35
		18.1			1.12	305.95
-Py			3.0		1.21	295.35
			3.4		1.25	298.65
			4.2		1.25	300.15
			5.0		1.30	302.95
-IBN				17.8	2.55	303.98

	55.8	1.82	303.55
	170.0	1.40	303.36
	213.0	2.31	303.37
	475.0	1.32	303.33
	45.8	2.18	303.34
-CONH$_2$[g]	93.1	1.13[h]	303.39
	143.6	1.18	303.34
	45.8	2.18	302.89
	93.2	1.22	303.09
	144.0	1.18	303.10
-CONH-Tr	170.0	1.17	303.33
	195.0	1.28	303.36
	202.0	1.56	303.34
	384.0	1.09	303.42

[a] $M_{n,th} = M_{NIPAM}[NIPAM]_0 conv/[initiator]_0$.

[b] Determined from ^{1}H NMR spectroscopy (500 MHz) in D_2O, 25 °C.

[c] Determined by MALDI–TOF–MS.

[d] Determined by light scattering in methanol.

[e] Determined by GPC calibrated with narrow-disperse polystyrene standards.

[f] Determined by turbidimetry, 50% transmittance (for samples of Xia *et al.* and Duan *et al.*) and onset point of the clouding curve (for samples of Furyk *et al.*).

[g] T_{cp} data were digitalized from original work of Furyk *et al.* using Origin 2018b [49]. The PDI of the corresponding samples before detritylation were used if the PDI of the samples after detritylation were not provided.

[h] The PDI of the sample after detritylation.

Table S2. T_{cp} versus M_n of PTEGSt containing various chain ends [20].

**H-PTEGSt-H**		**Ph-PTEGSt-H**		**H-PTEGSt-ON**		**Ph-PTEGSt-ON**	
**M_n (kDa)[a]**	**T_{cp} (K)[b]**	**M_n (kDa)[a]**	**T_{cp} (K)[b]**	**M_n (kDa)[a]**	**T_{cp} (K)[b]**	**M_n (kDa)[a]**	**T_{cp} (K)[b]**
3.1	301.16	3.9	285.17	3.0	284.12	3.9	279.18
5.7	294.64	6.6	285.66	5.7	287.12	6.6	281.16

8.3	293.15	8.2	288.13	8.3	287.12	8.2	283.67
11.1	293.15	14.4	290.64	11.1	289.18	14.4	286.71
12.5	293.18	22.9	290.68	12.5	289.14	22.9	287.60
16.7	293.18	26.4	289.78	16.7	290.15	26.4	288.13
28.0	290.64			28.0	289.21		

[a] Determined by GPC in THF, calibrated with standard monodisperse polystyrenes.

[b] Determined by turbidimetry: UV-Vis spectrometry (Method 1) and visualization technique (Method 2). $\triangle T_{cp}$ between two methods within 1 K.

Table S3. T_{cp} versus M_n of PHTrEGSt containing various chain ends [20].

**H- PHTrEGSt -H**		**H- PHTrEGSt -ON**		**Ph- PHTrEGSt -ON**	
**M_n (kDa)[a]**	**T_{cp} (K)[b]**	**M_n (kDa)[a]**	**T_{cp} (K)[b]**	**M_n (kDa)[a]**	**T_{cp} (K)[b]**
4.2	359.17	4.2	339.15	6.2	330.10
8.5	354.17	8.5	342.20	7.2	331.69
12.8	350.18	12.8	343.08	11.8	335.69
16.7	347.13	16.7	345.08	17.6	336.62
20.1	344.20	20.1	341.15	23.7	338.68
25.6	343.14	25.6	340.15		

[a] Determined by GPC in THF, calibrated with standard monodisperse polystyrenes.

[b] Determined by turbidimetry: UV-Vis spectrometry (Method 1) and visualization technique (Method 2). $\triangle T_{cp}$ between 2 methods within 1 K.

Table S4. R-groups (in original papers) versus corresponding end groups (in this work) in PNIPAM polymers [13,17,18,29].

**R-groups**	**M_R (kDa)**	**End groups**	**M_{E_1} (kDa)**
$-NH_2$	0.01602	**NH_2**$COCHCH_3$-	0.07209
-NH-*i*-Pr	0.05810	***i*-PrNH**$COCHCH_3$-	0.11417
-OMe	0.03103	**MeO**$COCHCH_3$-	0.08710
-OEt	0.04506	**EtO**$COCHCH_3$-	0.10112
-NHPh	0.09212	**PhNH**$COCHCH_3$-	0.14818
-Py	0.21724	**Py**$COCHCH_3$-	0.27330
-IBN	0.06810	**-IBN**	0.06810
$-CONH_2$	0.04403	**NH_2CO**$CH_2CH_2CCNCH_3$-	0.12515
-CONH-Tr	0.28635	**Tr-HNCO**$CH_2CH_2CCNCH_3$-	0.36746

S.2. The Invariance of T_{cp} with Molar Mass

The telechelic PNIPAM-*n*Bu studied by Qiu *et al.* shows a clear invariance of T_{cp} with molar mass [24]. As noted by the authors the limiting value of T_{cp} at high molar mass is different from values in literature. The cause for the slight difference is not clear. Therefore, we will not analyze the experimental data in ref [24] but use our PNIPAM homopolymer reference $\chi_{OM} = 3.1595 - 805.34/\mathrm{T}$ to create a hypothetical invariant end functionalized homopolymer for a concentration $\phi_{cp} = 0.05$ for which $\boldsymbol{\chi_{cp}}(\mathbf{M} \to \infty) = 0.517318$, $\boldsymbol{T_{cp}}(\mathbf{M} \to \infty) = 304.80$.

We discuss the invariance of the system based on Equation (6) in the manuscript and define $\chi_{1,\mathrm{inv}}$ and $\chi_{2,\mathrm{inv}}$ for the invariant case,

$$\begin{aligned}\chi = \chi_{OM} &+ \left(-\chi_{OM}M_E + \chi_{OE_1}M_{E_1} + \chi_{OE_2}M_{E_2} - \chi_{ME_1}M_{E_1} - \chi_{ME_2}M_{E_2}\right)\frac{1}{M} \\ &+ \left(\chi_{ME_1}M_E M_{E_1} + \chi_{ME_2}M_E M_{E_2} - \chi_{E_1E_2}M_{E_1}M_{E_2}\right)\frac{1}{M^2} \\ &= \chi_{OM} + \frac{\chi_{1,\mathrm{inv}}}{M} + \frac{\chi_{2,\mathrm{inv}}}{M^2}\end{aligned} \qquad (6')$$

with

$\chi_{1,\mathrm{invar}} = -\chi_{OM}M_E + \chi_{OE_1}M_{E_1} + \chi_{OE_2}M_{E_2} - \chi_{ME_1}M_{E_1} - \chi_{ME_2}M_{E_2}$ and

$\chi_{2,\mathrm{invar}} = \chi_{ME_1}M_E M_{E_1} + \chi_{ME_2}M_E M_{E_2} - \chi_{E_1E_2}M_{E_1}M_{E_2}$

For a single end group this reduces to

$\chi_{1,\mathrm{inv}} = -\chi_{OM}M_{E_1} + \chi_{OE_1}M_{E_1} - \chi_{ME_1}M_{E_1}$ and $\chi_{2,\mathrm{inv}} = \chi_{ME_1}M_{E_1}^2$

All χ_{IJ}, including $\chi_{1,\mathrm{inv}}$ and $\chi_{2,\mathrm{inv}}$ are in principle temperature functions. However, since Tcp is invariant with molar mass all χ's are just constants and cannot be determined independently from the invariant data. Therefore, we can only determine χ_{OM}, $\chi_{1,\mathrm{inv}}$ and $\chi_{2,\mathrm{inv}}$. We use Equations (6') and (7) directly to determine the parameters from the invariant data. The result is given in **Figure S1**.

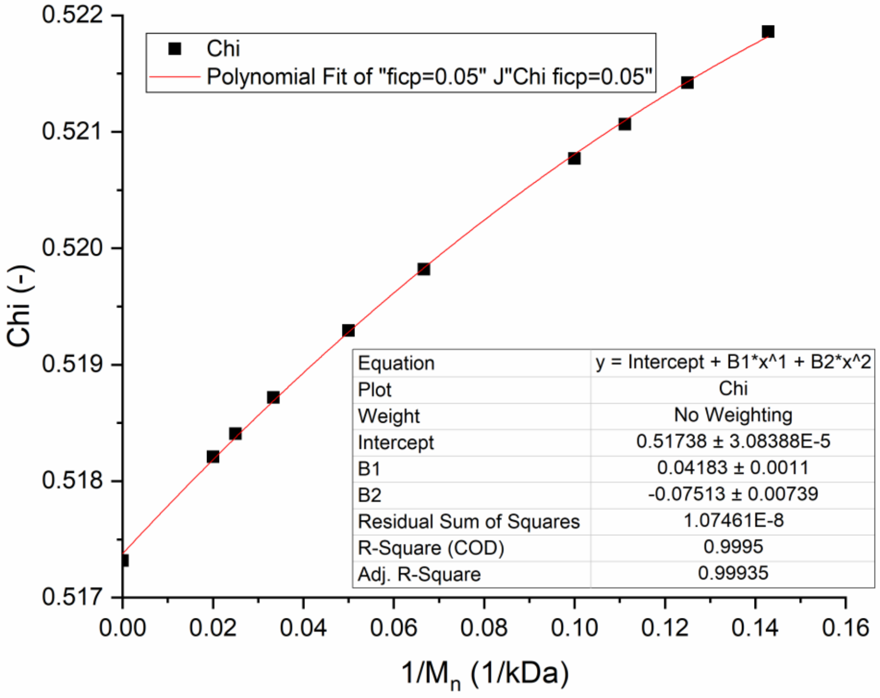

Figure S1. χ vs. $1/M_n$ for the hypothetical invariant end-functionalized PNIPAM system (■: χ values determined by Equation (7) for the hypothetical invariant T_{cp} values at 9 values of M, the value at $M \to \infty$ is $\chi_{cp}(M \to \infty)$, solid red line is the fit according to Equation (6′)). The fitted parameters for χ_{OM}, $\chi_{1,\text{invar}}$, and $\chi_{2,\text{invar}}$ are given in the table in the plot by respectively Intercept, B1 and B2.

From **Figure S1** it is clear that the simple FH theory for end-functionalized homopolymers can also predict or fit the invariance of T_{cp} with molar mass. We observe that invariance implies that the quadratic term in χ and $\Delta\chi$, defined in Equations (6′), (8) and (9), must be present. Moreover, invariance can be reached not only with 2 end group but also with 1 end group. Invariance results when $\chi_{2,\text{inv}}$ is sufficiently large to observe the necessary quadratic dependence on M^{-1} in χ and thus $\Delta\chi$.

For a single end group we can interpret the result as follows: the FH interaction parameter of the solvent middle segment, χ_{OM}, is positive and equal to the value for which the hypothetical homopolymer (no distinct end groups) has its T_{cp}, $\chi_{1,\text{inv}} = -\chi_{OM}M_{E_1} + \chi_{OE_1}M_{E_1} - \chi_{ME_1}M_{E_1} > 0$ and $\chi_{2,\text{inv}} = \chi_{ME_1}M_{E_1}^2 = -0.07513$ means that $\chi_{ME_1} < 0$, i.e. the middle – end segment pair interaction is energetically more favorable compared to the arithmetic average of the middle – middle and end group – end group pair interactions. To have $\chi_{1,\text{inv}} > 0$ and knowing that $-\chi_{OM}M_{E_1}$

and $\chi_{ME_1}M_{E_1}$ are both < 0, $\chi_{OE_1}M_{E_1}$ must be positive and $\chi_{OE_1}M_{E_1} > \chi_{1,\text{inv}} - \chi_{OM}M_{E_1} + \chi_{ME_1}M_{E_1}$.

Depending on the molar mass of the end group the values of χ_{OE_1} and χ_{ME_1}vary to produce the necessary compensation effect. As an example, say $M_{E_1} = \frac{1}{2}$kDa we get $\chi_{OE_1} = 0.30067$ and $\chi_{ME_1} = -0.30052$.

Also, for 2 distinct end groups a similar compensation must occur, but we cannot determine the numerical contribution of both end groups from the measurement of only the invariant end-functionalized homopolymer but measurement of e.g., at least 1 end-functionalized homopolymer carrying one of the 2 end groups will make it possible to separate the contributions of both end groups in the invariant end-functionalized homopolymer and in this manner quantify the compensation effect.

S.3. 0.95 Confidence Intervals and Values of t-Statistics and P-Values for the FH Parameter Values

S.3.1. Homopolymers

Parameters	Estimate	Standard Error	t-Statistic	P-Value
$chiOM_0$	3.15431	0.114874	27.4589	$2.18026*10^{-8}$
$chiOM_1$	-804.402	35.7112	-22.5252	$8.60153*10^{-8}$

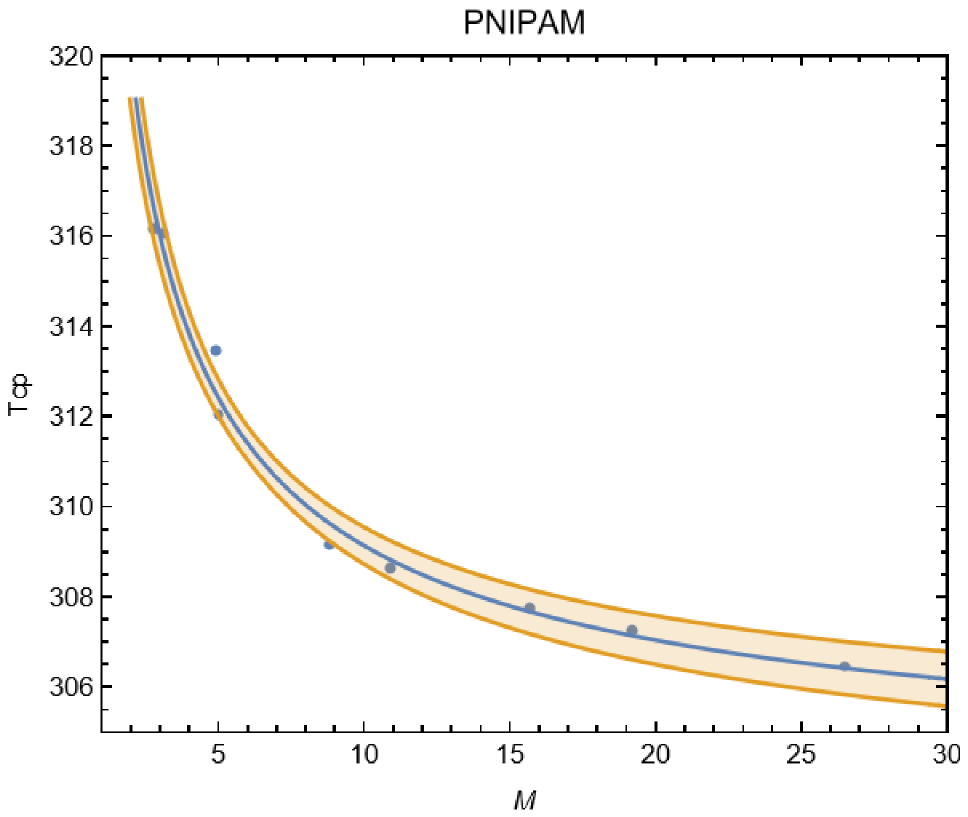

Parameters	Estimate	Standard Error	t-Statistic	P-Value
chiOM0	3.04525	0.344691	8.83473	0.000308671
chiOM1	-731.819	101.419	-7.21582	0.000796929

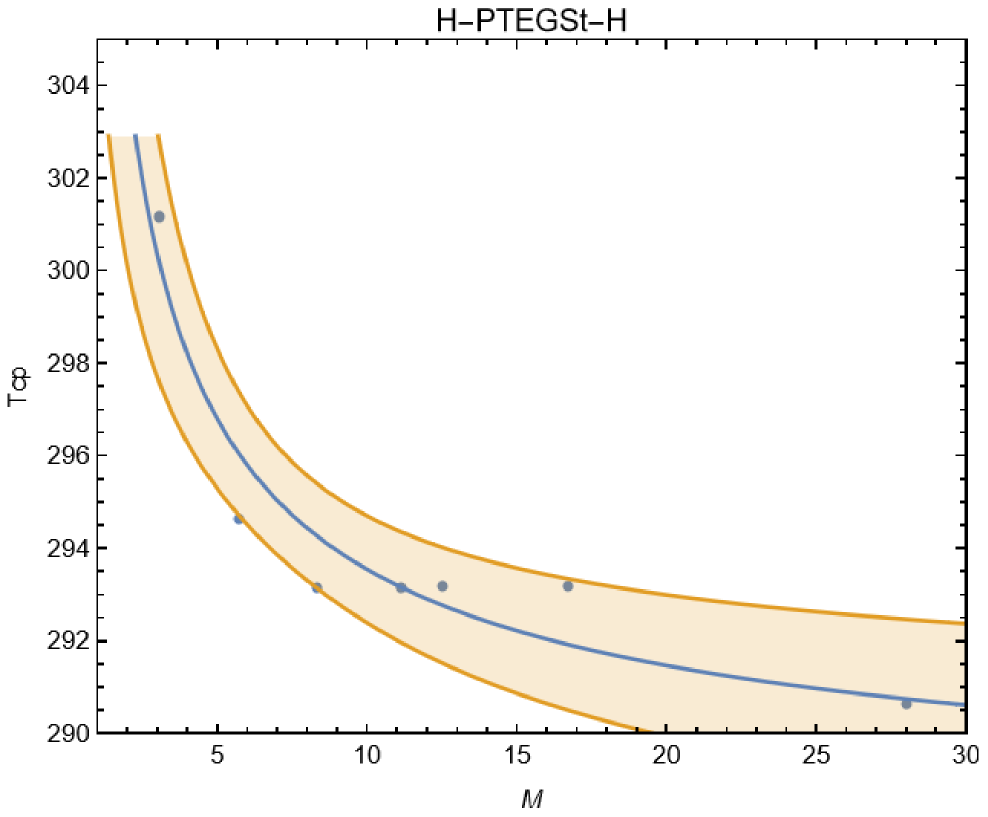

Parameters	Estimate	Standard Error	t-Statistic	P-Value
$chiOM_0$	1.84016	0.175305	10.4969	0.000465669
$chiOM_1$	-451.424	61.3352	-7.35994	0.00181555

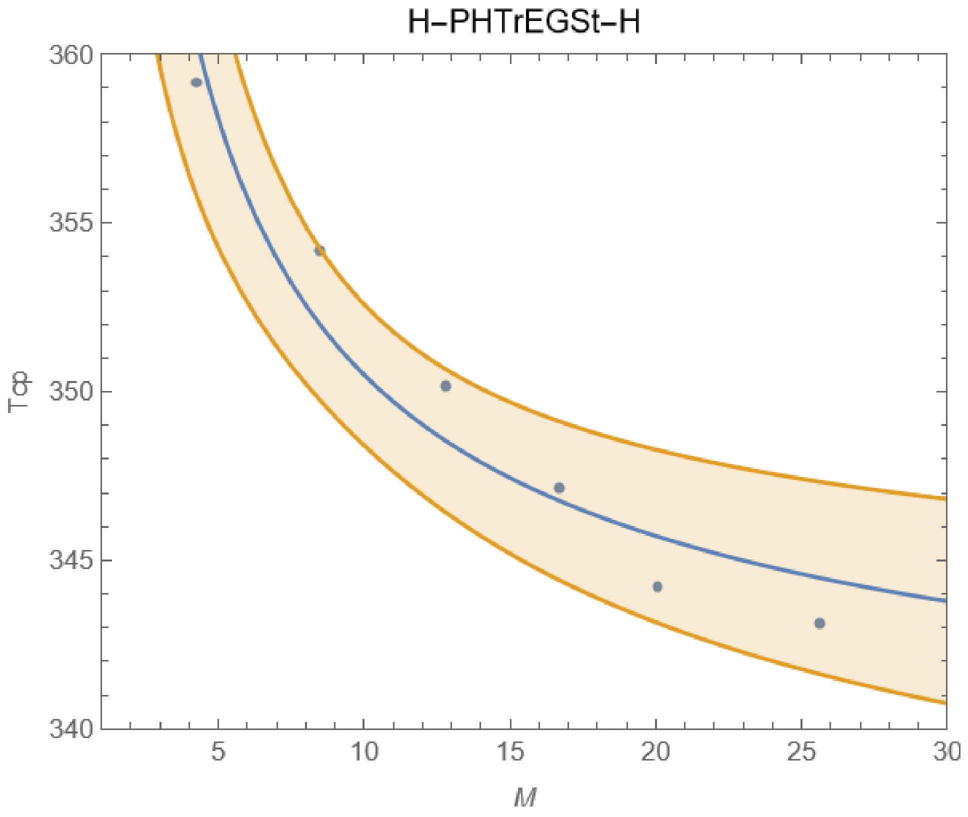

S.3.2. End functionalized homopolymers

Parameters	Estimate	Standard Error	t-Statistic	P-Value
chiOE1	-0.129933	0.126188	-1.02967	0.378913

Out[•]=

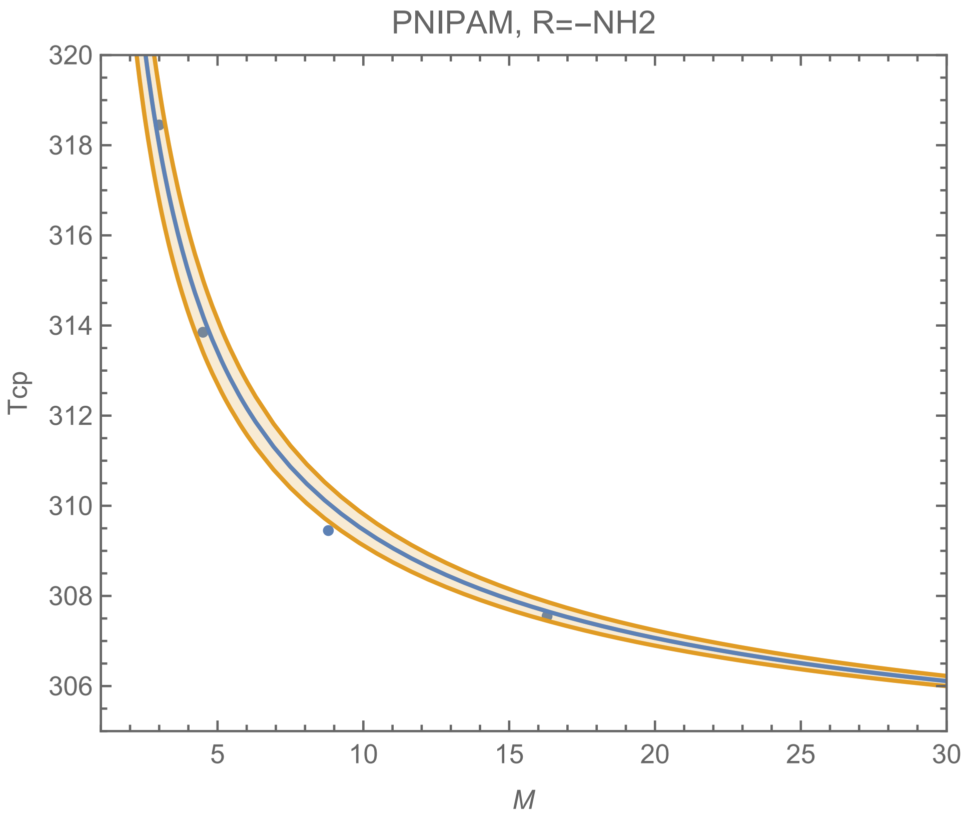

Parameters	Estimate	Standard Error	t-Statistic	P-Value
chiOE1	2.4567	0.151928	16.1702	0.000514493

Out[ ]=

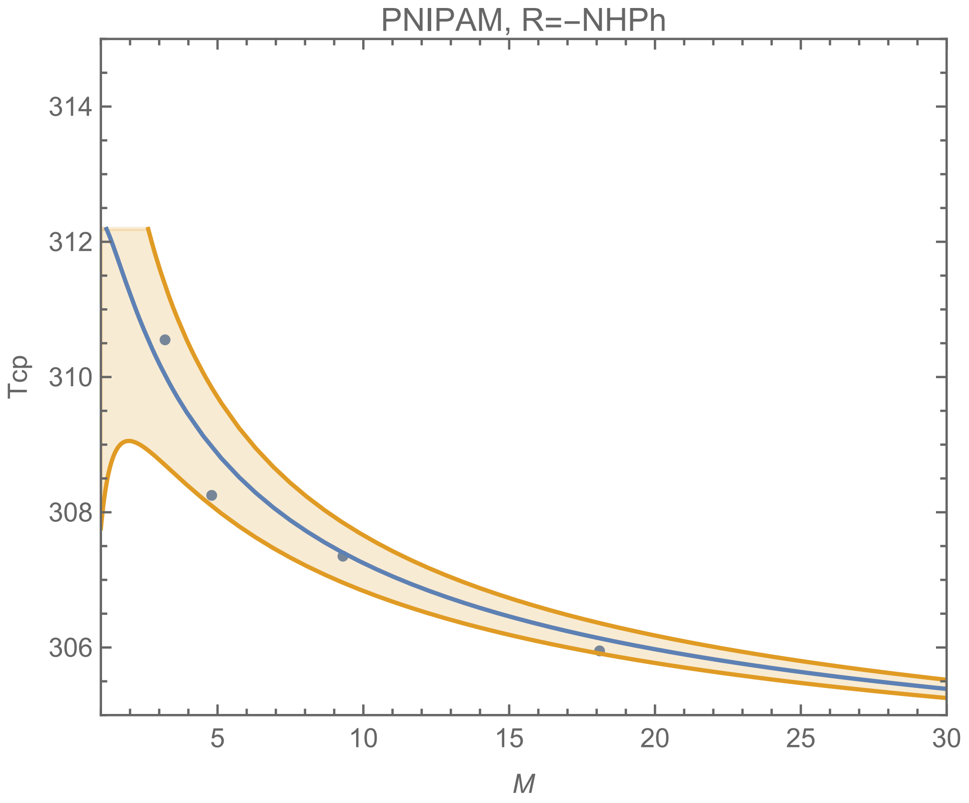

Parameters	Estimate	Standard Error	t-Statistic	P-Value
chiOE1	1.21289	0.120295	10.0826	0.00207769

Out[ ]=

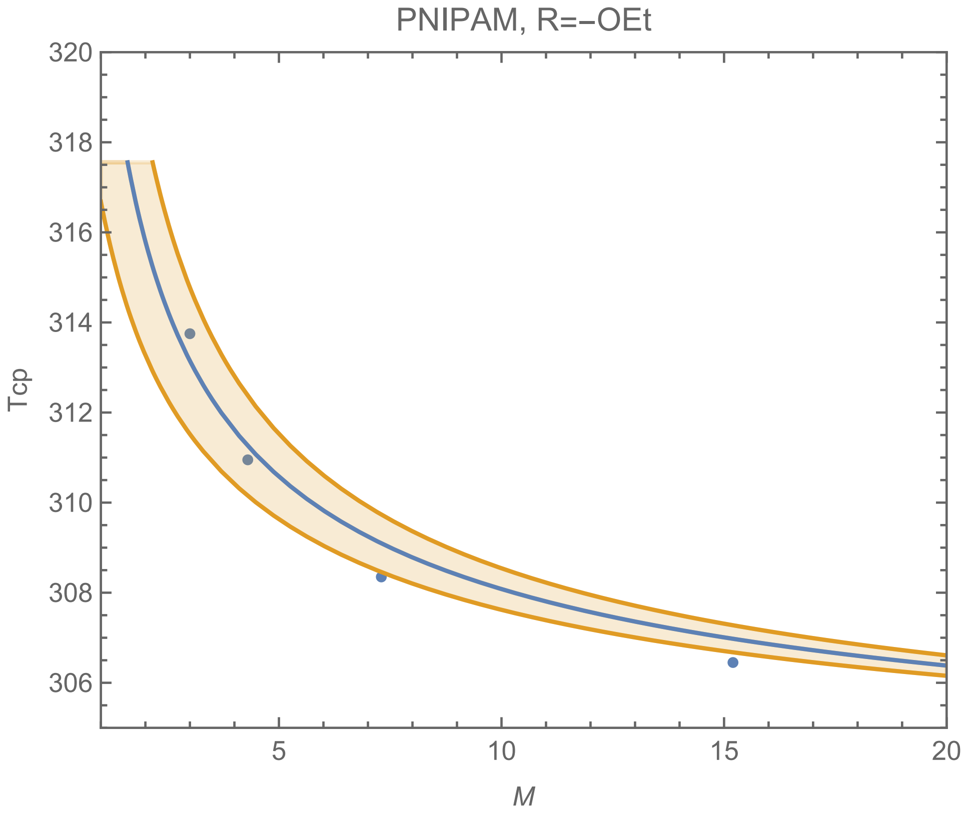

Parameters	Estimate	Standard Error	t-Statistic	P-Value
chiOE1	2.22604	0.0810532	27.464	0.000105952

Out[•]=

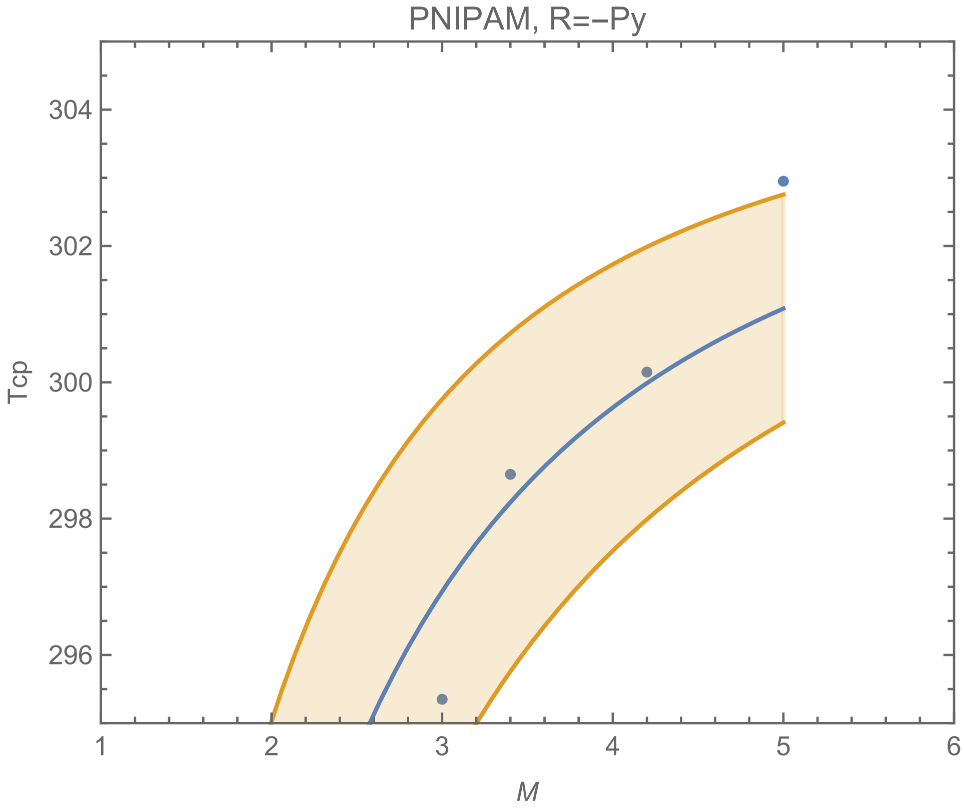

Parameters	Estimate	Standard Error	t-Statistic	P-Value
chiOE1	7.8893	0.614718	12.834	0.000212484

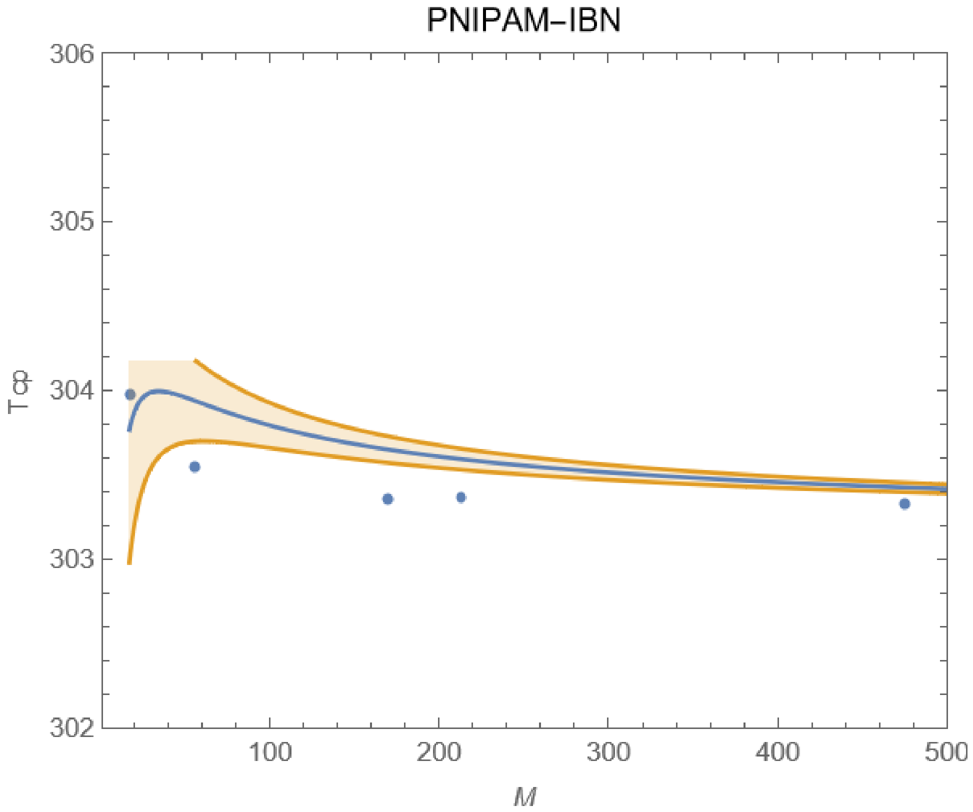

Parameters	Estimate	Standard Error	t-Statistic	P-Value
chiOE1	3.17644	0.105911	29.9917	$9.11412*10^{-8}$

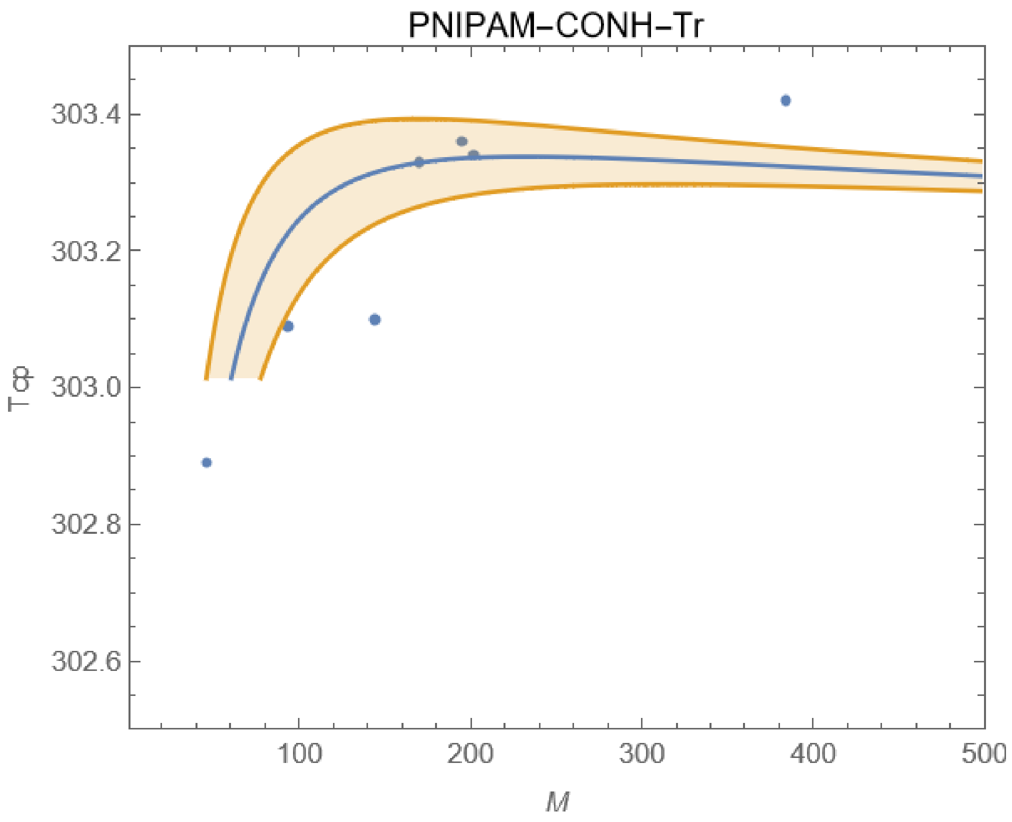

Parameters	Estimate	Standard Error	t-Statistic	P-Value
chiOE1	2.64949	0.109529	24.1899	0.00170459

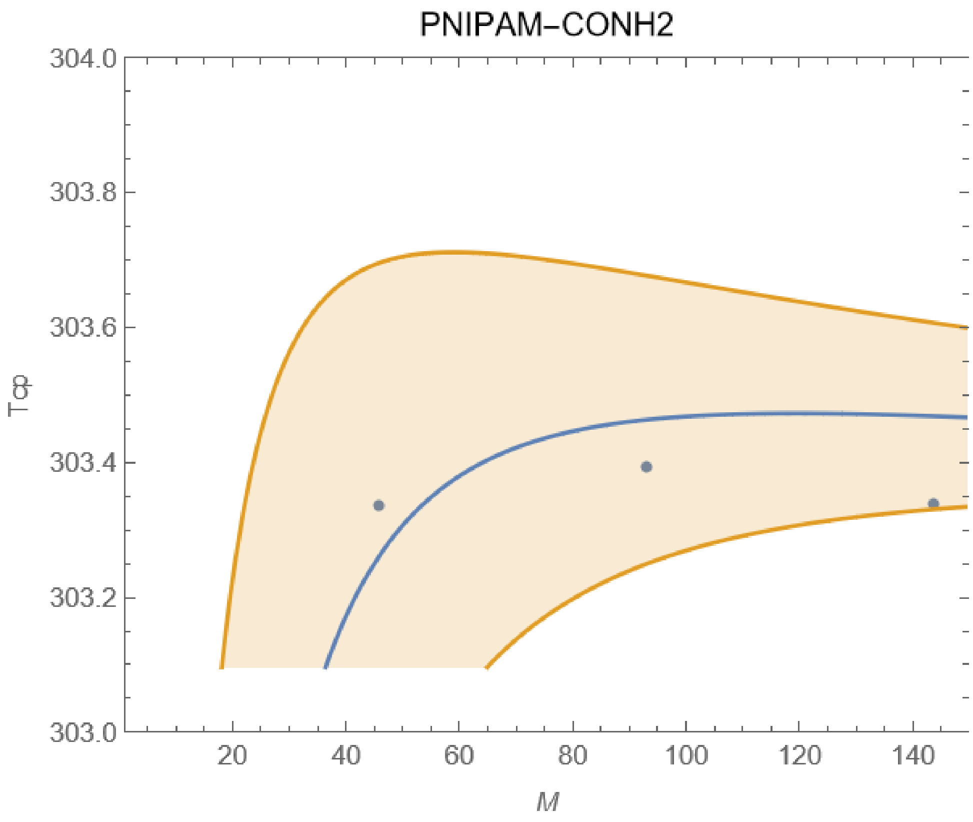

Parameters	Estimate	Standard Error	t-Statistic	P-Value
chiOE1	4.73893	0.363567	13.0345	0.0000474048

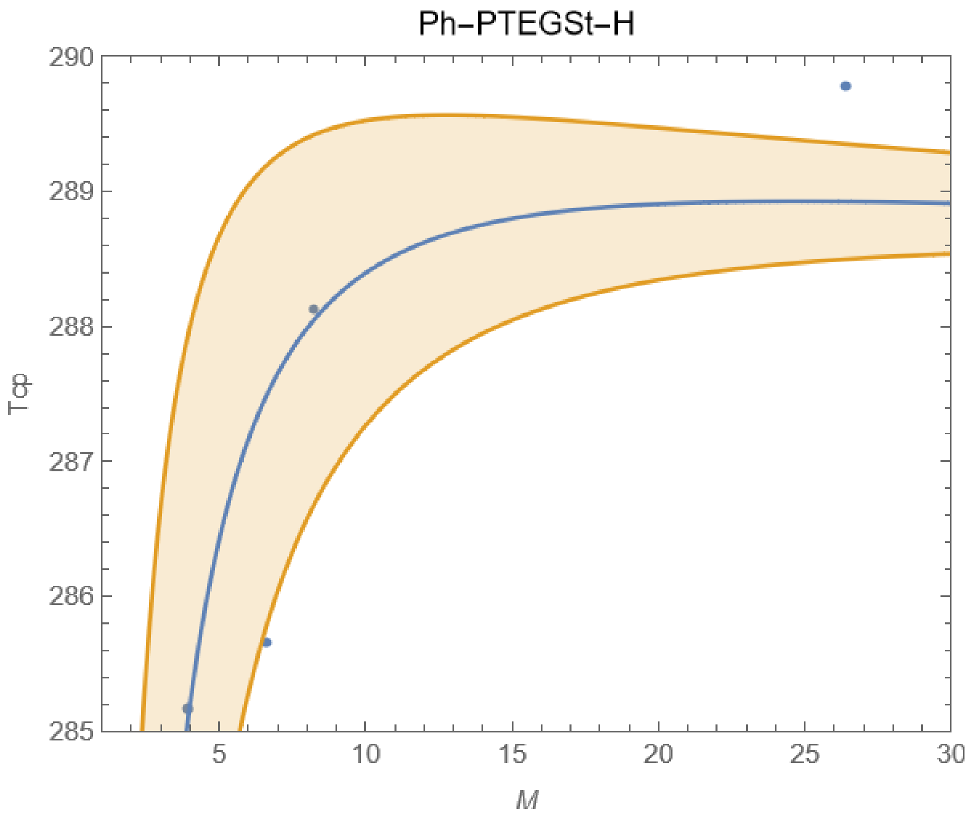

Parameters	Estimate	Standard Error	t-Statistic	P-Value
chiOE1	2.40779	0.0696248	34.5824	3.89467*10-8

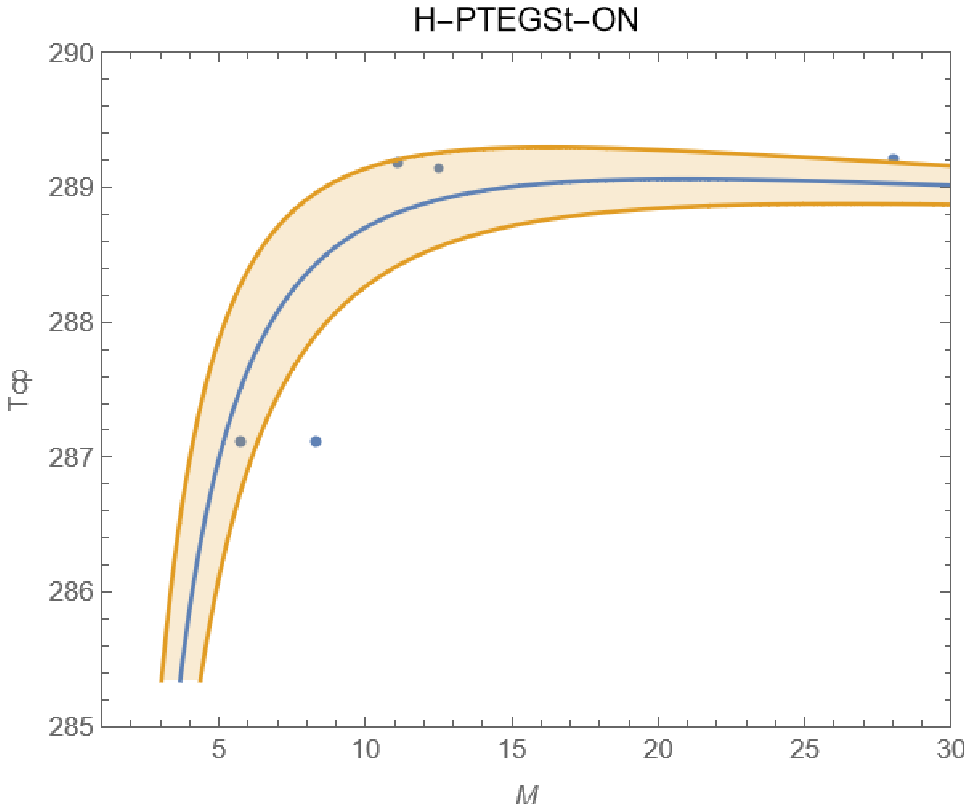

Parameters	Estimate	Standard Error	t-Statistic	P-Value
$chiOE_1$	4.81129	0.191407	25.1364	$1.85973*10^{-6}$

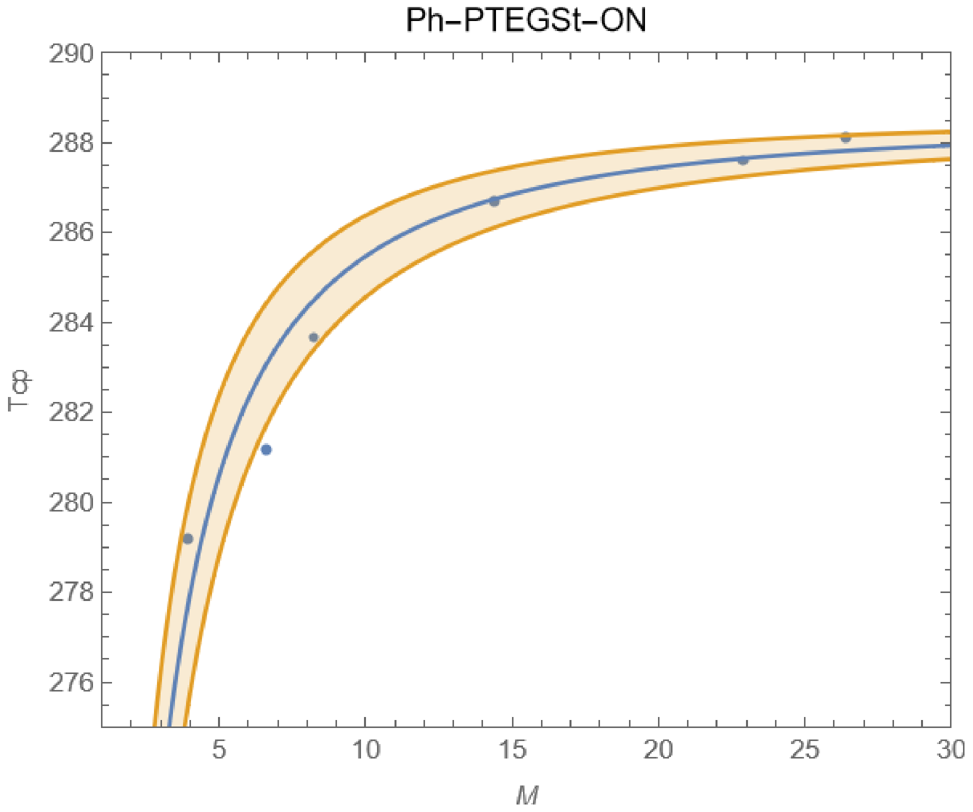

Parameters	Estimate	Standard Error	t-Statistic	P-Value
$chiOE_1$	2.46705	0.136594	18.0613	$9.55882*10^{-6}$

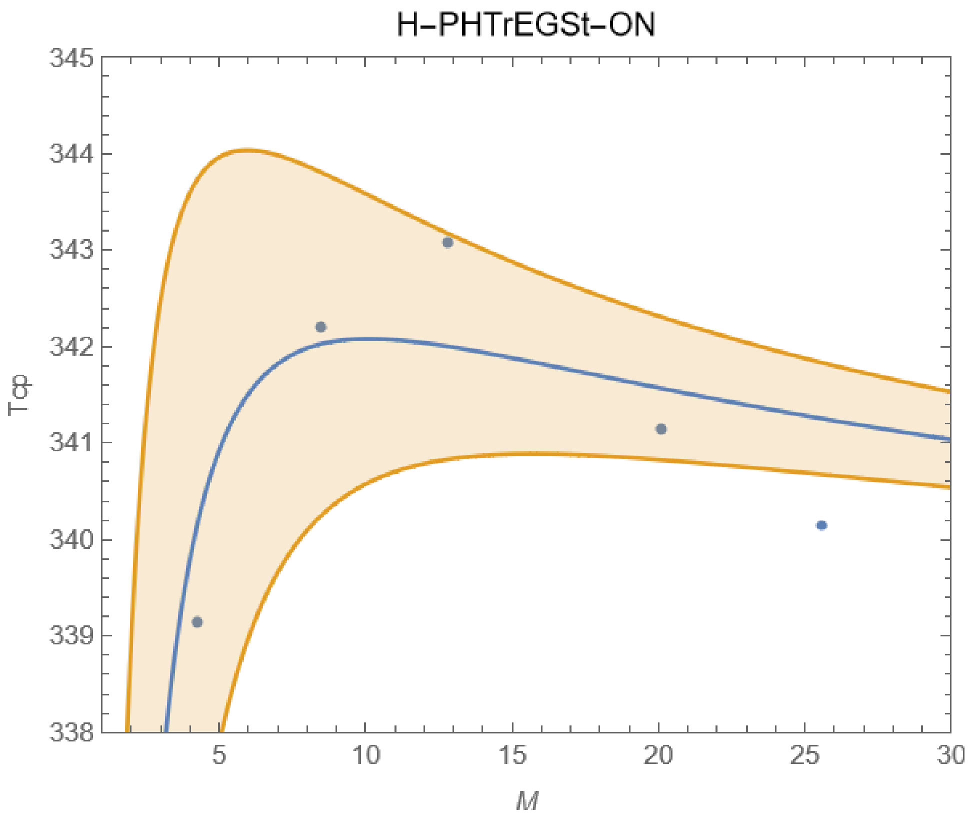

Parameters	Estimate	Standard Error	t-Statistic	P-Value
$chiOE_1$	3.24023	0.0409955	79.0388	$1.53578*10^{-7}$

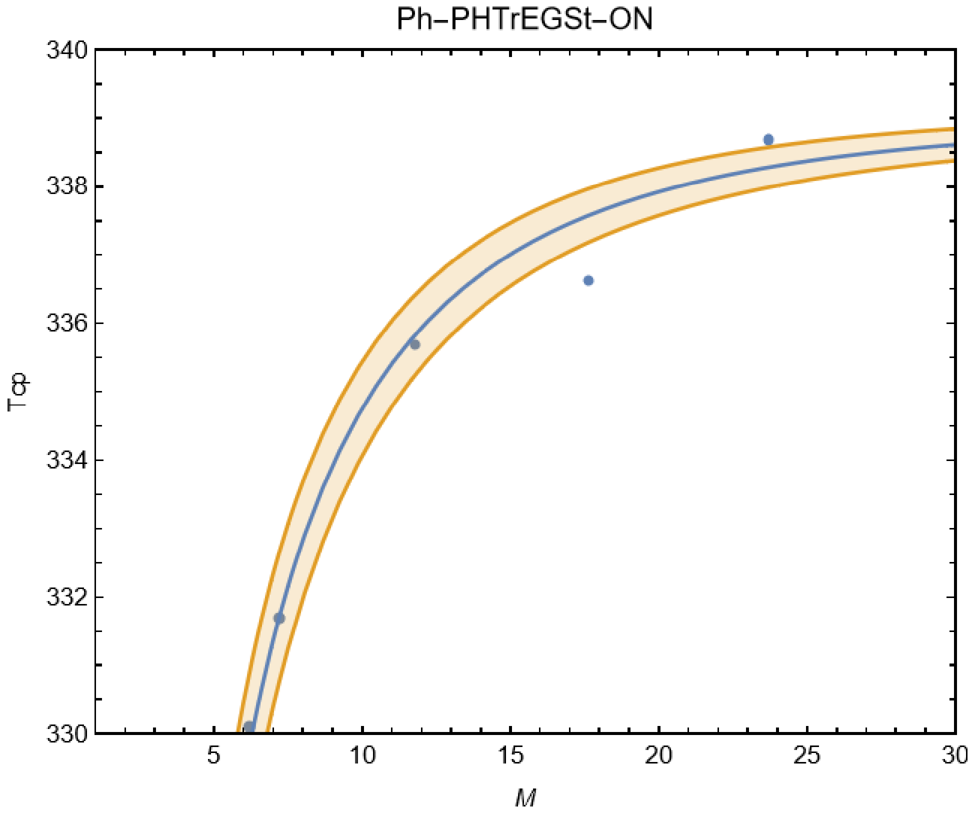

Supplement: Supplementary file 1 [file polymers-16-00563-s001.zip › polymers-2814296-supplementary.pdf]
